# Cisplatin-Induced Hearing Loss, Oxidative Stress, and Antioxidants as a Therapeutic Strategy—A State-of-the-Art Review

**DOI:** 10.3390/antiox13121578

**Published:** 2024-12-21

**Authors:** Olaf Rose, Tim Croonenberg, Stephanie Clemens, Tobias Hinteregger, Stefanie Eppacher, Petra Huber-Cantonati, Marta Garcia-Miralles, Raffaella Liuni, Silvia Dossena

**Affiliations:** 1 Institute of Pharmacy, Pharmaceutical Biology and Clinical Pharmacy, Paracelsus Medical University, 5020 Salzburg, Austriastephanie.clemens@pmu.ac.at (S.C.);; 2 Center of Public Health and Health Services Research, Paracelsus Medical University, 5020 Salzburg, Austria; 3 Institute of Pharmacology and Toxicology, Paracelsus Medical University, 5020 Salzburg, Austria; 4 Research and Innovation Center Regenerative Medicine & Novel Therapies (FIZ RM&NT), Paracelsus Medical University, 5020 Salzburg, Austria

**Keywords:** antioxidants, cisplatin, cisplatin-induced hearing loss (CIHL), hearing loss, ototoxicity, oxidative stress

## Abstract

Cisplatin is an established component of treatment protocols for various solid malignancies but carries a significant potential for serious adverse effects. Ototoxicity from cisplatin treatment is an important dose-limiting toxicity that manifests as bilateral, progressive, irreversible, dose-dependent sensorineural hearing loss, ear pain, tinnitus, and vestibular dysfunction. Despite the recent approval of sodium thiosulphate for the prevention of cisplatin-induced hearing loss (CIHL) in pediatric patients, structured prevention programs are not routinely implemented in most hospitals, and reducing platinum-induced ototoxicity in adults remains an important clinical problem without established treatment options. Cochlear oxidative stress plays a fundamental role in CIHL. Here, we review the molecular mechanisms leading to oxidative stress in CIHL and the clinical and preclinical studies testing antioxidants in CIHL to guide future clinical trials in assessing the efficacy and safety of candidate antioxidant compounds in this clinical setting.

## 1. Introduction

The platinum (Pt) drugs cisplatin, carboplatin, and oxaliplatin are established components in many treatment protocols for solid malignancies in adult and pediatric patients. Cisplatin and carboplatin are commonly used in regimens for small-cell lung cancers (SCLCs), localized non-small-cell lung cancer (NSCLC), and a variety of other malignancies, including breast, testicular, ovarian, cervical, prostate, head and neck, bladder, and refractory non-Hodgkin’s lymphomas [1,2]. Oxaliplatin is a platinum compound effective in metastatic colorectal cancer and as adjuvant therapy in colorectal cancer [3,4].

Though widely used and highly effective, Pt compounds have significant potential for serious adverse effects. Acute toxicities of cisplatin, such as nephrotoxicity, neurotoxicity, and ototoxicity, are chemotherapy dose-limiting toxicities and result in dose reductions, treatment delay, or treatment cessation in at least 30% of the treated patients [5,6]. According to WHO, there have been 20 million new cancer cases in 2022 worldwide (https://gco.iarc.fr/today/en, accessed on 20 November 2024). The exposure of an estimated one million of these patients to cisplatin and/or carboplatin would have resulted in approximately half a million cases of hearing loss [7]. As the incidence of new cancer cases is projected to be 35 million in 2050, which represents a 77% increase from 2022 (https://www.who.int/news/item/01-02-2024-global-cancer-burden-growing--amidst-mounting-need-for-services, accessed on 20 November 2024), the new cases of hearing loss due to platinum-based chemotherapy are predicted to escalate in parallel. The improvement of oncological treatments and the increased survival rate of cancer patients demand the identification of adequate measures to ameliorate their quality of life. As treatment modifications, including dose reduction or the choice of an alternative drug, are often not possible, and given the irreversible nature of hearing loss, there is an urgent need for preventive measures in this setting.

As detailed in the following, oxidative stress plays a fundamental role in ototoxicity induced by platinum compounds. Thus, the pharmacological use of antioxidants as otoprotectants appears logically justified. However, any otoprotectant must not interfere with the efficacy of the anticancer therapy and have an acceptable safety profile. Identifying the molecular and cellular targets of oxidative stress is fundamental in choosing an effective antioxidant and the route of administration. Other crucial factors are the dose and timing of administration. Here, we review the mechanism of cisplatin-induced oxidative stress and the clinical and preclinical studies on the use of antioxidants in cisplatin-induced ototoxicity to guide future clinical trials for the identification of the most effective and safe otoprotection.

## 2. Cisplatin Anticancer Mechanism of Action

Cisplatin is a water-soluble planar coordination complex containing a central Pt atom bound to two chlorine atoms and two amino (NH_3_) groups. Concerning their mechanism of action, cisplatin and Pt compounds are classically included in the group of crosslinking chemotherapeutics. The generalized mechanism of action involves four key steps: (i) cellular uptake, (ii) aquation/activation, (iii) DNA binding, and (iv) the cellular processing of DNA lesions. DNA binding involves the generation of nuclear DNA adducts with the formation of covalent bonds with purine DNA bases, leading to intrastrand and interstrand DNA cross-links. If not repaired, these DNA lesions cause cell death as a consequence of DNA replication and transcription blockage [2,8].

## 3. Cisplatin Ototoxicity

### 3.1. Clinical Features

Cisplatin-induced ototoxicity, manifested as sensorineural hearing loss, ear pain, tinnitus, and vestibular dysfunction (vertigo), has a negative long-term impact on multiple areas of life of patients and can compromise their quality of life [9].

Ototoxicity may occur within hours to days after treatment with cisplatin. Cisplatin-induced hearing loss (CIHL) is dose-related, cumulative, usually permanent, unilateral or bilateral, occurs initially in the higher frequencies (4 to 8 kHz), and may later progress to the lower frequencies, affecting the ability to hear a normal conversation [10]. Hearing loss can be severe and has also been observed after the initial cisplatin dose [11]. In the pediatric population, particularly in patients younger than 5 years, ototoxicity is more pronouced [12], and hearing loss might go unnoticed, affecting the learning process and social development.

According to cisplatin prescription information in Germany (https://www.fachinfo.de/fi/detail/012558/cisplatin-teva-r-1-mg-ml-konzentrat, accessed on 20 November 2024), “Careful monitoring with audiometry should be performed before initiating therapy and before administering further doses of cisplatin. (…) An audiogram should be obtained before starting treatment with cisplatin and before each new treatment cycle”. The USA prescription information recommends “consider audiometric and vestibular function monitoring in all patients receiving cisplatin for injection” (https://www.accessdata.fda.gov/drugsatfda_docs/label/2022/018057s092lbl.pdf, accessed on 20 November 2024). However, these recommendations are inconsistently implemented in the routine clinical practice. Obstacles include the incomplete awareness of the problem among clinicians, the underestimation of the impact of hearing loss on the quality of life of an oncological patient, and the absence of standardized assessment methods and reference values [13]. These obstacles also hamper the implementation of adequate preventive measures in clinical practice.

### 3.2. Incidence

Hearing loss occurs in a significant proportion of patients under cisplatin therapy and has been reported in 36% of adult patients and 40–60% of pediatric patients [13,14,15,16]. Other sources indicate an incidence of up to 75–100% of patients [17,18]. In adolescents and young adults defined as 15–39 years of age, moderate/severe CIHL was reported in 44% of the patients [19]. There are also concerns that in utero exposure to cisplatin or carboplatin can lead to hearing loss in childhood [20].

Among Pt regimens, cisplatin or the combination of cisplatin and carboplatin appears more ototoxic than carboplatin when given alone. For example, in the study of Knight et al., 55% of the children treated with cisplatin, 38% of the children treated with carboplatin, and 84% of the children treated with both agents developed sensorineural hearing loss [21]. Accordingly, global prevalence estimates in adults are higher for regimens involving cisplatin only (49.21%) and cisplatin plus carboplatin (56.05%) compared to carboplatin only (13.47%) [7]. With oxaliplatin, although rare, hearing loss can be sudden and severe [22,23].

While hearing loss is a well-recognized side effect of Pt compounds, tinnitus and vestibular dysfunction are less frequently assessed. The incidence of tinnitus was reported to be 31% and 12% with cisplatin and carboplatin, respectively [24]. The rate of abnormal objective vestibular function test results after chemotherapy administration varied from 0 to 41.7% among clinical studies [25]. Overall, the lack of harmonization of assessment methods and reference values and the variability of risk factors make assessing the magnitude of the problem difficult in this context.

### 3.3. Risk Factors

The risk of cisplatin ototoxicity is increased by several factors [26,27,28]:

- Cisplatin (cumulative) dose;
- Duration of therapy;
- Younger patient age;
- Older patient age;
- Noise exposure;
- Exposure to other ototoxic drugs;
- Depleted nutritional state;
- Cranial irradiation;
- Pre-existing hearing impairment;
- Family history of hearing loss;
- Tobacco use;
- Comorbidities;
- Renal impairment;
- Genetic predisposing factors.

Youngest patients (<5 years) and those with a brain tumor, hepatic tumor, or neuroblastoma had the highest prevalence of CIHL [12,19]. Concerning co-morbidities, diabetes, hypertension, and hypercholesterolemia have been identified as risk factors for ototoxicity, intended as hearing loss and/or tinnitus [27]. Concomitant exposure to other ototoxic drugs, such as vincristine, aminoglycoside antibiotics, or furosemide, represents an additional risk factor [12,19,29].

#### Genetic Factors Predisposing to Cisplatin Ototoxicity

Table 1 and Table 2 list the gene variants associated with cisplatin ototoxicity or otoprotection in original studies or meta-analyses. Well-established ototoxic SNPs fall in the genes *ACYP2*, *COMT*, and *TPMT*.

*ACYP2* encodes an acylphosphatase that can hydrolyze the phosphoenzyme intermediate of different membrane pumps, including the Ca^2+^/Mg^2+^-ATPase from the sarcoplasmic reticulum, and was initially thought to be specific to the skeletal muscle. *ACYP2* is also expressed in the cochlea, and an *ACYP2* mutation may potentially interfere with intracellular ATP-dependent Ca^2+^ signaling due to the disruption of Ca^2+^ homeostasis [30].

*COMT* encodes catechol-O-methyltransferase, an enzyme that catalyzes the breakdown of neurotransmitters such as dopamine, epinephrine, norepinephrine, and other catechol-containing compounds. The enzyme introduces a methyl group to the catecholamine, which is donated by S-adenosyl methionine (SAM). Thus, increased levels of SAM consequent to enzyme malfunction could become ototoxic in association with cisplatin [31]. The levels of neurotransmitters norepinephrine and dopamine may also play a role. Norepinephrine modulates the central auditory system at several levels, and dopaminergic neurons are part of lateral olivocochlear complex-derived efferent fibers. Also, adrenergic receptors have recently been found within the cochlea [32,33]. More studies are needed to understand whether *COMT* mutations interfere with these pathways.

*TPMT* encodes the enzyme thiopurine methyltransferase, which methylates thiopurine compounds. The methyl donor is again SAM. Thus, the susceptibility to cisplatin following *TPMT* mutations can also be related to increases in the concentration of SAM, as suggested for *COMT* [31].

Given the prominent role of oxidative stress in cisplatin ototoxicity and the fundamental role of the endogenous antioxidant system in CIHL, it is not surprising that genes like *GSTP1*, *GSTT1*, *NFE2L2*, and *SOD* are involved in susceptibility to cisplatin ototoxicity (see Section 4.2.3). Glutathione S-transferases (GSTs) are a family of isozymes that catalyze the conjugation of the reduced form of glutathione (GSH) to xenobiotic substrates, including cisplatin, for the purpose of detoxification. *NFE2L2* encodes the transcription factor nuclear factor E2-related factor 2 (Nrf2), which regulates the transcription of numerous ROS-detoxifying enzymes such as glutathione peroxidase 2 (Gpx2) and several GSTs [34].

Curiously, some SNPs have been associated with both cisplatin ototoxicity predisposition and protection (Table 1 and Table 2). The reason for this discrepancy is unclear and warrants further studies. However, a risk linked to SNPs in *ACYP2* and *TPMT* appears plausible (Table 1). 

As detailed in Section 5, the only approved drug for the prevention of cisplatin-induced ototoxicity is systemic sodium thiosulfate, which can be used in children and adolescents with non-metastatic cancers. In the authors’ opinion, patients belonging to this specific patient group who also carry SNPs predisposing to cisplatin ototoxicity in *ACYP2* and *TPMT* should be prioritized for pharmacological otoprotection with systemic sodium thiosulfate during cisplatin therapy. According to an expert panel, evaluating the effectiveness of sodium thiosulfate administration in patients with genetic susceptibility to cisplatin-induced ototoxicity is a substantial gap in knowledge and deserves investigation [35].

**Table 1:** Genetic factors predisposing to cisplatin ototoxicity.

Gene	SNP ID	Reference
*ACYP2*	rs1872328	[30,36,37,38,39,40,41]
*COMT*	rs4646316	[42]
*COMT*	rs9332377	[31,39,41,43]
*CTR1*	rs10981694	[44]
*GSTP1*	rs1695	[45,46]
*GSTT1*	null	[47,48]
*LRP2*	rs2075252	[39,49]
*NFE2L2*	rs6721961	[48]
*SLC47A1*	rs2289669	[50]
*SOD*	rs4880	[39,51]
*TPMT*	rs1142345	[39,52]
*TPMT*	rs12201199	[31,39,52]
*TPMT*	rs1800460	[39,52]
*XPC*	rs2228001	[41,53]

SNP, single nucleotide polymorphism.

**Table 2:** Genetic factors protecting against cisplatin ototoxicity.

Gene	SNP ID	Reference
*ABCB5*	rs10950831	[39]
*ABCC3*	rs1051640	[39,52]
*COMT*	rs4646316	[39,41]
*EPXH1*	rs2234922	[39,54]
*GSTM1*	null	[48,55]
*GSTM3*	rs1799735	[39,56]
*GSTP1*	rs1695	[41] [47,57] *
*GSTT1*	null	[43,58] *
*LRP2*	rs2228171	[58] *
*NFE2L2*	rs6721961	[39,59]
*Otos*	rs77124181	[60]
*Otos*	rs2291767	[60]
*SLC16A5*	rs4788863	[61]
*SLC22A2*	rs316019	[39,59,62]
*XPD/ERCC2*	rs1799793	[41] [47] *

SNP, single nucleotide polymorphism. * WT allele ototoxic.

## 4. Oxidative Stress-Dependent Cellular and Molecular Mechanisms of Cisplatin-Induced Ototoxicity

### 4.1. Tissue and Cellular Targets of Oxidative Stress

Oxidative damage has been observed following exposure to cisplatin in several tissues, explaining the toxic effects on non-proliferating (non-cancer) cells and suggesting a role for oxidative stress in the pathogenesis of cisplatin-induced toxicities [63]. Oxidative stress, and specifically glutathione (GSH) depletion or inactivation and reactive oxygen species (ROS) of mitochondrial origin, has been established as an important factor that contributes to cisplatin nephrotoxicity, which is a major dose-limiting toxicity of this anticancer agent [64]. Similarly, cochlear ROS generation also plays a major role in cisplatin-induced ototoxicity [65]. It is unknown whether these effects are caused by the accumulation of cisplatin within the inner ear structures or by a higher sensitivity of the inner ear tissue to cisplatin. As a matter of fact, cisplatin is indefinitely retained within the cochlea after chemotherapy, prolonging the exposure of sensitive targets to the offending agent [66].

**Figure 1:**
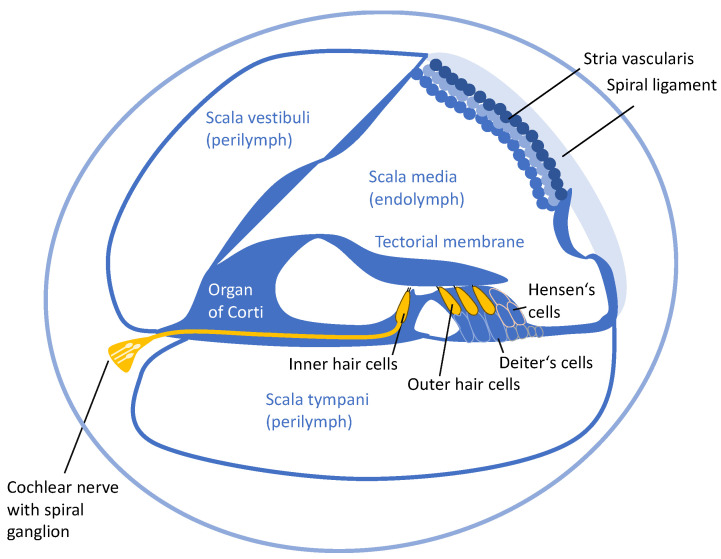
Schematic representation of a cross-section of the cochlea.

Inner ear structures established to be affected by cisplatin are the organ of Corti, the stria vascularis, the spiral ligament, and the spiral ganglia (Figure 1). Cisplatin can enter the inner ear via the blood vessels of the stria vascularis, cross the blood–lymph barrier, and reach the endolymph. Once in the endolymph, cisplatin can enter the sensory hair cells of the organ of Corti through various molecular entities, including copper transporter 1 (CTR1), organic cation transporter-2 (OCT2), mechanoelectrical transduction channel (MET), and transient receptor potential (TRP) channels [67]. Then, cisplatin damages hair cells by generating intracellular ROS, depleting the GSH stores, and leading to lipid peroxidation and protein S-nitrosylation [68,69,70]. These changes, directly linked to oxidative stress, represent an important factor in determining hair cell loss, and outer hair cells (OHCs) of the basal and second turns of the cochlea are more sensitive to this effect. However, the morphological alterations of Hensen’s cells and Deiter’s cells appear even earlier than those involving sensory hair cells [71]. Thus, oxidative stress probably involves both sensory cells and supporting cells, but the latter, although affected, seems to be more resistant [72]. Another hallmark of cisplatin ototoxicity in the organ of Corti is the loss of ribbon synapsis between the inner hair cells and primary afferent auditory neurons, which might be linked to oxidative/nitrative stress [73]. The fact that a natural antioxidant prevented these changes in an animal model of cisplatin-induced ototoxicity [74] (Supplemental Table S1) supports this hypothesis.

The stria vascularis, which is a highly metabolically active structure responsible for the generation and maintenance of the endocochlear potential, shows a particularly high accumulation of cisplatin compared to other inner ear structures [66], becomes edematous after exposure to cisplatin [75], and shows excessive accumulation of platinum-DNA adducts in the marginal cells [76]. Protein S-nitrosylation, secondary to nitrosative stress, involves all turns of the stria vascularis and the spiral ganglion [70]. The oxidative stress-dependent loss of Na^+^/K^+^ ATPase alpha subunit in the stria vascularis and spiral ligament was also observed [74]. These changes are consistent with the loss of function of the stria vascularis and the consequent fall of the endocochlear potential observed in animal models of cisplatin ototoxicity [66,77].

Mitochondrial ROS accumulation, decreased activity of superoxide dismutase (SOD), and increased malondialdehyde (MDA) content were also detected in the pericytes of the stria vascularis of mice exposed to cisplatin [78], which might affect the blood–labyrinth barrier integrity and amplify the leakage of cisplatin into the endolymph, further promoting toxicity at the level of the organ of Corti.

A complex chain of events occurring as a consequence of or in parallel to oxidative stress, including cytokine release and inflammation, direct DNA damage, and mitochondrial and endoplasmic reticulum dysfunction, lead to programmed cell death in the target tissues via various processes, including intrinsic apoptosis, necroptosis, autophagy, pyroptosis, and ferroptosis [67,79]. Thus, OHC loss and fall in the endocochlear potential consequent to stria vascularis dysfunction, possibly in conjunction with cochlear synaptopathy, appear to be the main consequences of oxidative stress leading to CIHL.

Oxidative stress and mitochondria dysfunction causing neuronal apoptosis have been proposed as determinants of cisplatin-induced neurotoxicity [63]. Therefore, in principle, the central auditory pathways could also be affected by cisplatin-induced oxidative stress and contribute to hearing loss. More studies are needed to verify this hypothesis.

### 4.2. Molecular Determinants of Oxidative Stress

The main molecular mechanisms leading to oxidative stress after the exposure of inner ear tissues to cisplatin include the activation of nicotinamide adenine dinucleotide phosphate (NADPH) oxidase 3 (NOX3) and xanthine oxidase (XO), inhibition of glutathione peroxidases (Gpx), and abatement of the endogenous antioxidant system.

#### 4.2.1. Nicotinamide Adenine Dinucleotide Phosphate (NADPH) Oxidases (NOX)

NOX3 is an isoform of NOX that is prominently, although not exclusively, expressed in the inner ear in the cochlea and catalyzes the NADPH-dependent reduction of oxygen to form superoxide (O_2_^•-^) [80].

In NOX3-transfected HEK 293 cells, Banfi et al. first showed that pre-incubation with cisplatin markedly enhanced superoxide production [81]. In the rat cochlea and UB/OC-1 cells, a conditionally immortalized cell line derived from the mouse cochlea, cisplatin treatment increased Nox3 expression and ROS production [82,83]. These studies show that cisplatin can directly stimulate and upregulate the biosynthesis of NOX3.

After the exposure of mice to cisplatin, Nox3 expression showed a robust increase in the supporting cells within the organ of Corti and spiral ganglion neurons of the basal turns of the cochlea [84]. These observations point to the supporting cells as a source of cochlear ROS. Delivering these ROS to the OHC and subsequent lipid peroxidation would cause the apoptosis and loss of OHC, ultimately leading to hearing loss.

Together, these findings establish NOX3 as a main molecular determinant of ROS production in the inner ear after exposure to cisplatin. Accordingly, ABR threshold shifts showed no deterioration, OHC and spiral ganglion neuron apoptosis were lower, and OHC loss was reduced in Nox3-deficient mice exposed to cisplatin [84]. Importantly, the transtympanic injections of Nox3 siRNAs inhibited the cisplatin-induced apoptosis of OHC, stria vascularis, and spiral ganglion neurons and prevented CIHL in rats [85,86]. These studies identify NOX3 as a promising therapeutic target in CIHL.

NOX3 does not appear to be the only NOX isoform induced by cisplatin. Treatment with cisplatin also induced the expression of NADPH oxidase isoforms NOX1 and NOX4 in HEI-OC1 auditory cells and in vivo. The pharmacological inhibition of NADPH oxidases or RNA interference for NOX1 and NOX4 abolished ROS production and protected viability in cisplatin-treated cells [87]. Whether NOX1 and NOX4 can be valid therapeutic targets in CIHL awaits further studies.

#### 4.2.2. Xanthine Oxidase (XO)

The enzyme xanthine oxidase (XO) is active in the cochlea, contributes to the generation of ROS such as O_2_^•-^ and H_2_O_2_, and is upregulated by cisplatin [88]. XO converts hypoxanthine to xanthine and xanthine to uric acid and is inhibited by the uricostatic drug allopurinol. The combination of allopurinol and ebselen, a glutathione peroxidase (Gpx) mimetic and peroxynitrite scavenger, reduced cisplatin-induced ototoxicity in rats [75] (Supplemental Table S1).

#### 4.2.3. The Endogenous Antioxidant System

The endogenous antioxidant system, represented by antioxidant enzymes catalase (CAT) and SOD, as well as endogenous GSH, GSH-regulating enzymes, and other enzymes, is altered following cisplatin exposure. SOD catalyzes the dismutation of the superoxide (O_2_^•-^) anion radical into molecular oxygen (O_2_) and hydrogen peroxide (H_2_O_2_). CAT catalyzes the decomposition of H_2_O_2_ to H_2_O and O_2_. Glutathione peroxidases (Gpxs) mainly catalyze the reaction of H_2_O_2_ and 2GSH to form GSSH and 2H_2_O. In the presence of NADPH, GSH reductase restores reduced glutathione (GSH) from its oxidized GSSG form. The thioredoxin redox system protects thiols from oxidation and consists of reduced thioredoxin (TRX) that catalyzes the reduction of disulfides (–S–S–), becoming oxidized and subsequently reduced by thioredoxin reductase (TrxR), another NADPH-consuming enzyme [89,90].

As previously mentioned, cisplatin can be inactivated following binding to GSH via an enzymatic reaction catalyzed by glutathione-S-transferases (GST). Thus, GSH plays a fundamental role in cisplatin toxicity and resistance [91,92]. Cisplatin ototoxicity in rats, manifested as a fall in the endocochlear potential and increased ABR threshold, correlated with significantly elevated malondialdehyde levels, depletion in cochlear GSH, and decreased activities of Gpx and GSH reductase. The activities of cochlear SOD and CAT were also altered compared to untreated animals [93,94,95,96]. Accordingly, a significant decrease in the transcript levels of the *Sod1* and *Gpx1* genes in the cochlea and a decreased Gpx1 immunoreactivity specific to the stria vascularis were detected [97]. Thus, decreased activity and/or expression of key antioxidant enzymes is an important factor in cisplatin-induced ototoxicity, with Gpx playing a fundamental role. Accordingly, ebselen, a Gpx mimetic, conferred otoprotection in rats exposed to cisplatin when given alone or combined with allopurinol [75,96] (Supplemental Table S1). A recently discovered compound with Gpx-like activity protected from cisplatin-induced ototoxicity in cochlear explants and after oral administration in mice [98] (Supplemental Table S1). The inhibition of TrxR may be a contributing factor in cisplatin-induced ototoxicity [99].

#### 4.2.4. Mitochondria

In porcine proximal tubular cells, cisplatin inhibited the complexes I and IV of the mitochondrial transport chain that, in association with the inhibition of the GSH-regenerating enzyme GHS reductase, contributed to ROS formation [100]. Cisplatin has been shown to damage the mitochondria in the cochlear organ of Corti-derived cell line HEI-OC1 and cause mitochondrial membrane potential collapse [101]. Thus, the direct partial inhibition of the mitochondrial electron transport chain with consequent ROS formation and other mechanisms of mitochondrial dysfunction, including the mutation of mitochondrial DNA and lipid peroxidation of mitochondrial membranes, play a role in cisplatin ototoxicity [102,103].

#### 4.2.5. Aldose Reductase

Aldose reductase is a cytosolic enzyme that catalyzes the NADPH-dependent conversion of glucose or galactose to sorbitol. Sorbitol is impermeant to cell membranes, and its accumulation in the intracellular environment contributes to osmotic stress and the formation of advanced glycation end (AGE) products. An initial study showed no otoprotection from an aldose reductase inhibitor in a model of galactose-induced ototoxicity [104]. However, an interesting recent study showed prominent aldose reductase transcript, protein, and activity upregulation in cochlear explants exposed to cisplatin. These effects were accompanied by NADPH and GSH depletion, the upregulation of antioxidant enzymes, and cytosolic and mitochondrial ROS production and lipid peroxidation [105]. The upregulation of aldose reductase leads to the exhaustion of NADPH, which is an essential cofactor for GSH reductase, and consequently leads to GSH depletion, exacerbating oxidative damage and cellular dysfunction. The inhibition of aldose reductase with the flavonoid tiliroside reduced cisplatin-induced toxicity in HEI-OC1 cells, cochlear hair cells, and spiral ganglia ex vivo. Transtympanic tiliroside and systemic epalrestat, an aldose reductase inhibitor approved for the treatment of diabetic neuropathy, alleviated cisplatin-induced cochlear degeneration and hearing loss in mice [105].

## 5. Clinically Approved Antioxidant Drugs for the Prevention of Cisplatin-Induced Ototoxicity

Given the frequent use of cisplatin and other platinum compounds in cancer patients and the high rate of ototoxicity, strategies to reduce the risk of platinum-induced ototoxicity in the clinical setting are highly warranted. Systemic sodium thiosulfate (STS), a thiol-class otoprotectant, demonstrated efficacy in two international phase 3 trials (ACCL0431 [106] and SIOPEL6 [107]) and is the only currently approved drug to prevent CIHL in children (Table 3). However, nearly a third of patients receiving STS in the two trials still developed CIHL, and STS did not offer protection from cisplatin-induced nephrotoxicity or myelosuppression, it may have exacerbated cisplatin nephropathy, and STS infusions have been complicated by infusion-related reactions, including vomiting, hypotension, and/or severe rigors. Importantly, in ACCL0431, the 3-year overall survival was significantly lower for treatment with sodium thiosulfate than for observation in children with disseminated disease, indicating that STS interferes with the anticancer efficacy of cisplatin and precluding its use in this population [35,108]. In SIOPEL 6, which enrolled children with standard-risk (localized) hepatoblastoma, the overall survival or event-free survival was not affected. No drugs are approved for use in the adult.

Regarding the use of systemic sodium thiosulfate for the prevention of cisplatin-induced ototoxicity in children and adolescents with cancer, a panel of experts made a strong recommendation for administration in non-metastatic hepatoblastoma, a weak recommendation for administration in other non-metastatic cancers, and a weak recommendation against its routine use in metastatic cancers. Amifostine, sodium diethyldithiocarbamate, and intratympanic therapy should not be routinely used, and cisplatin infusion duration should not be altered as a means to reduce ototoxicity according to this expert panel [35].

## 6. Clinical Trials Testing Antioxidant Pharmacological Strategies for the Prevention of Cisplatin-Induced Ototoxicity

Several drugs have been tested in preclinical studies for the prevention of platin-induced hearing loss during the past decades (Table S1). However, only a few clinical trials have been conducted (Table 3). The main approach is the systemic application of a drug, either orally or intravenously, as opposed to transtympanic application. Despite its cumbersome administration, transtympanic use combines the general advantages of high concentrations on the site of action with a low probability of systemic interference with the chemotherapy. A frequent and convenient study approach was the treatment of one ear with the other ear serving as a control.

### 6.1. N-Acetylcysteine

N-acetylcysteine (NAC) is a thiol compound that can act as a direct free radical scavenger and increase intracellular GSH levels. The seminal study showing NAC otoprotection against cisplatin dates to 1997 and was performed in postnatal day 3 rat explants of the organ of Corti. The mean hair cell density and stereocilia bundle integrity were morphologically evaluated. The addition of 10^−3^ mol/L NAC to the medium 24 h before and during a 48 h exposure to 6–12 µg/mL cisplatin provided significant protection against ototoxicity [68]. Later, several preclinical studies confirmed a significant otoprotection of NAC against cisplatin-induced damage (Table S1). The clinical studies are detailed below and in Table 3.

#### 6.1.1. Intravenous N-Acetylcysteine

In a recent clinical trial, intravenous N-acetylcysteine decreased the likelihood of CIHL in children at the end of cisplatin therapy and recommendations for hearing interventions at the end of the study. Infusion reactions were also common in this study [118]. N-acetylcysteine was effective in adults in a small study by Yildirim et al. at doses of 600 mg/day along with cisplatin therapy. Hearing loss was prevented at 10,000 and 12,000 Hz with N-acetylcysteine [122].

These and the two clinical trials described in Section 5 provided the proof of concept that intravenous antioxidants can be valuable otoprotectants in CIHL (Table 3). Notably, three out of four trials have been conducted in patients < 21 y. Tinnitus and vestibular dysfunction were not evaluated in these trials. A trial by Dósa et al. explored safety and found no toxicity for i.v. and i.a. N-acetylcysteine doses up to 450 mg/kg in stage 3 renal failure patients [131]. Efficacy was not assessed in this study.

#### 6.1.2. Transtympanic N-Acetylcysteine

Three studies evaluated the efficacy of transtympanic N-acetylcysteine (Table 3). In the study by Yoo et al., [119] N-acetylcysteine was applied transtympanically to one ear, with the other ear serving as a control during cisplatin therapy. Only two out of eleven patients showed improved hearing at ≥4 or ≥8 kHz at 3 months from baseline, and the study did not reach statistical significance. Riga et al. administered N-acetylcysteine to 20 patients during the hydration phase preceding cisplatin cycles [120]. The other ear was used as a control. Transtympanic injections showed significant effects on auditory thresholds at 8 kHz. Sarafraz et al. compared transtympanic injections of N-acetylcysteine or dexamethasone in a randomized clinical trial [121]. Auditory thresholds at 8 kHz increased at dexamethasone therapy but not at N-acetylcysteine, indicating the efficacy of N-acetylcysteine.

### 6.2. Sodium Thiosulfate

Sodium thiosulfate (STS) is an inorganic salt and a free radical scavenger with proven efficacy against cisplatin nephrotoxicity [132]. In an early study in guinea pigs, the co-administration of STS (1600 mg/kg/day i.p.) prevented click-evoked ABR deterioration following the i.m. administration of 1.5 mg/kg/day cisplatin for 8 days [133]. More recent preclinical studies with STS are included in Table S1. The clinical studies are detailed in Table 3 and below.

#### 6.2.1. Intravenous Sodium Thiosulfate

A less significant hearing threshold deterioration in patients with head and neck cancer receiving intra-arterial high-dose cisplatin chemoradiation plus STS was observed compared to patients receiving intravenous high-dose cisplatin chemoradiation without STS [134,135]. The design of these studies prevented predicting whether STS or the intra-arterial administration of cisplatin was the protective factor.

There are two additional well-designed clinical trials on this subject. The reader should refer to Section 5.

#### 6.2.2. Transtympanic Sodium Thiosulfate (STS)

Of the three trials on transtympanic STS, one assessed safety and two efficacy (Table 3). Transtympanic STS was tested for the prevention of cisplatin-induced ototoxicity in a randomized-controlled study by Duinkerken et al. [123]. STS was applied to one ear in 12 patients before cisplatin therapy. Eight of these patients developed hearing loss, which was milder in the pre-treated ear. The study did not reach significance, probably due to the low number of included patients. A study by Rolland et al. showed a non-significant trend toward hearing protection by transtympanic STS [124]. However, only three out of 13 patients completed all three cycles of treatment with STS.

### 6.3. Amifostine

Amifostine (WR2721) is an organic thiophosphate and free radical scavenger initially developed to protect normal tissue from radiation damage and later proposed to protect from cisplatin-induced nephrotoxicity following seminal experiments in Fischer rats [136]. Although early trials showed no efficacy, a small phase I escalation dose non-controlled trial supported otoprotection in patients with locally advanced cervical carcinoma treated with cisplatin and radiation therapy [137].

Three trials investigated the efficacy of amifostine i.v. on hearing protection in children undergoing cisplatin therapy (Table 3). Katzenstein et al. tested amifostine in children with hepatoblastoma [110]. There was no detectable difference compared to placebo in this study. Hypocalcemia was found as a side effect. In contrast, Gurney et al. found some protective effects against serious hearing loss in a large but non-randomized study on 263 children with average-risk medulloblastoma [111]. However, this was not true for high-risk patients. Accordingly, in the study of Fouladi et al., amifostine was effective in protecting children with average-risk medulloblastoma from severe ototoxicity. Also, this study was not randomized [109].

### 6.4. Vitamins

#### 6.4.1. Vitamin E

Vitamin E is a well-known antioxidant and was tested as a single agent against cisplatin-induced ototoxicity in a seminal study conducted by Teranishi et al. in guinea pigs [69]. Other preclinical studies investigated vitamin E alone or in combination with various agents (Table S1).

In clinical trials, vitamin E was tested for hearing protection during cisplatin therapy in a study by Villani et al. conducted in adult patients with solid malignancies [130] (Table 3). The study was designed as a randomized controlled trial with 54 patients assigned to the vitamin E arm and 54 patients assigned to the placebo arm. Unfortunately, only 13 and 10 patients completed the trial, respectively. The study detected a drop in hearing function at 2000 Hz and 8000 Hz at 1-month follow-up only in the control group, indicating the efficacy of oral vitamin E 400 mg/day in the prevention of cisplatin-induced hearing loss.

#### 6.4.2. Complex (Vitamin) Mixtures

In preclinical studies, antioxidant mixtures were often more effective than single agents in protecting from cisplatin-induced ototoxicity ([138] and Table S1).

Scasso et al. examined the protective effect of a combination of Q10, vitamins B1, B2, B6, B12, and E, choline, melatonin, *Ginkgo biloba* extract, and milk protein hydrolysate in randomized adult patients undergoing cisplatin therapy for various malignancies. The incidence of tinnitus was significantly lower in the verum than in the control group. Audiometry revealed better hearing ability at 8000 Hz for the right ear in this group. The collective was small, with 18 supplemented patients and eight patients as a control. A pitfall of this study was the missing effect in the left ear. It is impossible to link the effect to one of the ingredients [128] (Table 3).

### 6.5. Atorvastatin and Other Statins

Statins are a class of drugs approved to lower plasma levels of low-density lipoprotein (LDL) cholesterol by inhibiting 3-hydroxy-3-methylglutaryl-CoA (HMG-CoA) reductase, a rate-limiting enzyme in cholesterol synthesis. These drugs also possess antioxidant activities and proved effective in animal models of age-related and noise-induced hearing loss [139,140]. Fernandez et al. showed that lovastatin, which can cross the blood–brain barrier and potentially the blood–labyrinth barrier, reduced cisplatin-induced OHC and hearing loss in adult mice [141].

The same research group analyzed data from adult patients undergoing cisplatin chemotherapy for head and neck cancer regarding concomitant statin use [126] (Table 3). It was found that patients with statins had fewer incidences of hearing loss than patients who did not take a statin. This interesting observation was especially true for patients on atorvastatin, regardless of the dose. Due to the study design, causality between atorvastatin use and hearing protection cannot be concluded. However, this study indicates that further research should be carried out in this field.

### 6.6. Dexamethasone

The first study exploring the possible protective effect of dexamethasone on cisplatin-induced ototoxicity was conducted in guinea pigs, and dexamethasone was given intratympanically 30 min before cisplatin was given i.p. [142]. Other preclinical studies investigated dexamethasone in combination with other compounds (Table S1).

A randomized-controlled clinical study on intratympanic dexamethasone for the prevention of cisplatin-induced hearing loss was conducted by Marshak et al. [115]. Even though the study size was low, with only 15 patients included, a significant deterioration of pure-tone audiometry thresholds at 6000 Hz was found only in the control group. Other studies failed to show the efficacy of transtympanic dexamethasone (Table 3).

### 6.7. D-Methionine

D-methionine (D-Met) is a sulfur-containing compound, and its potential otoprotective effect in cisplatin-induced ototoxicity was first tested in rats. D-Met (75, 150, or 300 mg/kg) was given i.p. 30 min prior to cisplatin infusion and significantly preserved ABR threshold and OHC count [143]. Several other preclinical studies followed (Table S1).

A randomized controlled trial compared the hearing protection of oral D-methionine and placebo during cisplatin-based chemotherapy in adults. The D-methionine group (n = 14) showed no shift in auditory thresholds at 10, 11.2, and 12.5 kHz compared to the placebo group (n = 13). D-methionine was tolerated well by the patients [114] (Table 3).

### 6.8. Ginkgo Biloba Extract

Flavonoids and terpenoids like ginkgolides and bilobalide possess antioxidant and neuroprotective effects [144,145]. The *Ginkgo biloba* extract is approved for use against tinnitus in Germany.

The seminal study exploring the use of *Ginkgo biloba* extract in cisplatin-induced ototoxicity was performed in Japan. The authors showed that the extract, given 90 min before cisplatin, protected auditory thresholds and OHC in the Fisher rat. Importantly, the extract had no effect on the tumor growth rate in rats inoculated subcutaneously with SCC-158 squamous cell carcinoma cells, indicating that the extract did not interfere with the anticancer effect of cisplatin [146]. Several subsequent studies confirmed otoprotection (Table S1).

A study by Dias et al. explored a *Ginkgo biloba* extract applied orally at 240 mg/day during cisplatin therapy. The study was conducted as a randomized controlled trial with only 15 patients. Patients in the control group showed smaller distortion product otoacoustic emission (DPOAE) mean amplitudes and signal/noise ratio than patients who took *Ginkgo biloba* [117] (Table 3).

## 7. Preclinical Studies on Antioxidant Pharmacological Strategies for the Prevention of Cisplatin-Induced Ototoxicity

A comprehensive search has been conducted to identify effective antioxidant drugs against platinum compound-induced hearing damage in preclinical studies of the last 20 years.

### 7.1. Melatonin

Melatonin, the hormone produced by the pineal gland, is a pleiotropic biofactor that plays receptor-mediated and receptor-independent physiological functions, including regulating the circadian rhythm and antioxidant activity [147]. In various cell-based and animal models of oxidative stress-related diseases, melatonin directly neutralized free radicals and reactive oxygen and nitrogen species and restored the activity of the endogenous antioxidant enzymes SOD, Gpx, and GSH reductase [148,149,150,151,152,153,154].

In clinical trials, melatonin intake increased the total antioxidant capacity, SOD, GSH, Gpx, and GSH reductase activities and reduced malondialdehyde and protein carbonylation levels [155,156,157].

Concerning preclinical studies on the use of melatonin in drug-induced hearing loss, a recent systematic review with meta-analysis showed that melatonin can reduce the decrease in otoacoustic emission amplitude and provide protection against the ototoxic effects of cisplatin and aminoglycoside antibiotics at 5 kHz, 6 Hz, and 8 Hz in rats [158]. This review included the following three studies on cisplatin (Supplemental Table S1):

De Araujo et al. administered a 10 mg/kg single-dose cisplatin i.p. in the control and experimental groups. The dose of melatonin used in the experimental group was 1 mg/kg/day, i.p. [159].

Demir et al. used 12 mg/kg cisplatin in the control and experimental groups for five days. Intratympanic administration of melatonin (0.1 mg/mL) was carried out 30 min before the application of cisplatin in the experimental group [160].

Lopez-Gonzalez et al. administered a 10 mg/kg single-dose cisplatin i.p. in two groups: (1) oral melatonin in drinking water (10 mg/L) and (2) 250 µg melatonin s.c. daily seven days before the administration of cisplatin until the time of the experiment’s conclusion [161].

To the best of our knowledge, the pharmacological use of melatonin was never tested in patients with hearing loss, including CIHL.

### 7.2. Various Antioxidant Drugs and Substances Tested for the Prevention of Cisplatin-Induced Ototoxicity

A comprehensive search identified several antioxidant drugs and herbal products that have been tested against hearing loss during platinum-based chemotherapy. These studies are shown in the Supplemental Table S1. Intensively studied are alpha lipoic acid (seven studies), the carotenoid pigment astaxanthine (five studies), curcumin (six studies), and resveratrol (nine studies). All these drugs show strong antioxidant effects [162,163,164,165] and are potential candidates for further clinical research.

Resveratrol, a natural polyphenol found in grapes and red wine, has been shown to increase the endogenous antioxidant defense system in cochlear cells, thereby protecting them from cisplatin-induced damage. Resveratrol exerts anti-inflammatory effects, which can help mitigate the inflammatory response triggered by cisplatin in the inner ear and reduce the expression of pro-inflammatory cytokines like IL-6 and IL-1β [166]. Resveratrol also upregulates microRNA-455-5p, which modulates the PTEN-PI3K-AKT signaling axis to reduce oxidative stress in hair cells. Astaxanthine reduced ROS overexpression, mitochondrial dysfunction, and apoptosis mainly via the Nrf2-mediated pathway [167]. Curcumin’s effects on CIHL are also attributed to the reduction in oxidative stress. Additionally, it was shown that curcumin upregulated heme oxygenase-1 (HO-1), an enzyme that plays a role in cellular defense against oxidative stress [168].

Other antioxidants tested in CIHL are 5,7-Dihydroxy-4-methylcoumarin (D4M), allicin, allopurinol, apocynin, astragalosides, berberine, bucillamine, caffeic acid, carnitine, chrysin, copper, dexpanthenol, dieckol, DMSO, edaravone, epigallocatechin, erdosteine, ergothioneine, ethyl pyruvate, eugenol, eupatilin, ferrostatin, ferulic acid, formononetin, forskolin, fucoidan, fursultiamine, genistein, geranylgeraylacetone, ginseng, glutathione, hesperidin, hydrogen, hydroxytyrosol, isotretinoin, itaconate, KPR A020 (1,2,3 triazole derivate), lactate, levosimendan, lutein, lycopene, lysine-specific demethylase 1 inhibitors, mannitol, *Maytenus ilicifolia*, methylthiobenzoic acid, misoprostol, molsidomine, naringin, nicotinamide, nobiletin, nuciferine, oxytocin, peanut sprout, phycocyanin, polydatin, pomegranate, puerarin, quercetin, riluzole, Ringer’s lactate, salvianolic acid, schisandrin, selenium, silymarin, tempol, thiamine, thymoquinone, ursolic acid, vitamin C, whortleberry, and zingerone. The corresponding studies are listed in Supplemental Table S1 [74,75,78,94,96,98,101,158,159,160,162,166,167,168,169,170,171,172,173,174,175,176,177,178,179,180,181,182,183,184,185,186,187,188,189,190,191,192,193,194,195,196,197,198,199,200,201,202,203,204,205,206,207,208,209,210,211,212,213,214,215,216,217,218,219,220,221,222,223,224,225,226,227,228,229,230,231,232,233,234,235,236,237,238,239,240,241,242,243,244,245,246,247,248,249,250,251,252,253,254,255,256,257,258,259,260,261,262,263,264,265,266,267,268,269,270,271,272,273,274,275,276,277,278,279,280,281,282,283,284,285,286,287,288,289,290,291,292,293,294,295,296,297,298,299,300,301,302,303,304,305,306,307,308,309]. 

## 8. Conclusions and Future Directions

The fundamental contribution of oxidative stress in CIHL and the molecular targets leading to oxidative stress at the level of the cochlea following exposure to cisplatin are well established. More studies are needed to explore the possible involvement of retro-cochlear targets, like the ascending auditory pathways.

Given the crucial role of oxidative stress in CIHL, the clinical use of antioxidants as otoprotectants appears justified. Although STS i.v. has been approved for children, there are no established treatment options for adults. NAC i.v. was tested both in children and adults. More studies are needed to compare STS and NAC concerning safety and efficacy and to establish the best application route (i.v. vs. transtympanic). Oral agents, including statins and vitamin E, appear to be particularly attractive, but large prospective trials are needed to confirm efficacy. In designing new trials, more attention should be given to tinnitus and vestibular dysfunction in addition to hearing loss.

From preclinical studies, melatonin, lipoic acid, astaxanthine, curcumin, and resveratrol are potential candidates for clinical research.

## Figures and Tables

**Table 3:** Clinical studies testing antioxidants in cisplatin-induced hearing loss.

Study: First Author, Year	Study Design	Number of Assessed Participants	Interventional Drug, Application Mode	Platinum Compound	Primary or Auditory Endpoint	Result on Endpoint
Fouladi et al., 2008 [109]	NRCT	n = 62 amifostine; n = 35 control	Amifostine, i.v.	Cisplatin	Grade III or IV ototoxicity (>25 dB hearing loss in one ear) determined by conventional or conditioned PTA thresholds at 2000 Hz	Amifostine was effective in children with average-risk medulloblastoma
Katzenstein et al., 2009 [110]	RCT	n = 37 amifostine; n = 45 control	Amifostine, i.v.	Cisplatin/Carboplatin	Audiometry or ABR at 1–12 kHz	Amifostine was not effective
Gurney et al., 2014 [111]	NRCT	n = 328 amifostine; n = 51 control	Amifostine, i.v.	Cisplatin	Ototoxicity according to Chang grading scale [112] based on air (0.25–8 kHz) or bone (0.25–4 kHz) conduction by PTA, ABR (0.5–8 kHz), ASSR (0.5–8 kHz), or DPOAE (1–8 kHz) depending on patient status	Amifostine protected from serious hearing loss (Chang grade ≥2b) average-risk but not high-risk medulloblastoma patients
Crabb et al., 2017 [113]	RCT	n = 39 aspirin plus omeprazole; n = 40 control	Aspirin (acetylsalicylic acid), orally	Cisplatin	Combined hearing loss (cHL) * at 6 kHz and 8 kHz assessed by PTA	Aspirin was not effective.
Campbell et al., 2022 [114]	RCT	n = 14 D-methionine; n = 13 control	D-methionine, orally	Cisplatin	Auditory thresholds at 8, 10, 11.2 and 12.5 kHz	Auditory threshold shifts at 10, 11.2, and 12.5 kHz were observed with placebo; D-methionine prevented these shifts
Marshak et al., 2014 [115]	RCT	n = 15 (one ear treated, other ear control)	Dexamethasone, intratympanic	Cisplatin	Pure tone thresholds at 500, 1000, 2000, 3000, 4000, 6000, 8000 Hz; pure tone average (PTA) at 500–3000 and 4000–8000 Hz; DPOAE SNR and DPOAE SNR average at 1000–3000 Hz and 4000–8000 Hz	Intratympanic dexamethasone protected pure tone thresholds at 6000 Hz, DPOAE SNR at 7031 and 8391 Hz, and DPOAE SNR averages at 4000–8000 Hz
Moreno et al., 2022 [116]	RCT	n = 23 (one ear treated, other ear control)	Dexamethasone, transtympanic	Cisplatin	Auditory thresholds at 125, 250, 500, 1000, 2000, 3000, 4000, 6000, 8000 Hz assessed by PTA	Transtympanic dexamethasone was not otoprotective
Dias et al., 2015 [117]	RCT	n = 8 *Ginkgo biloba* extract; n = 7 control	*Ginkgo biloba* extract, 240 mg/day, orally	Cisplatin	DPOAE amplitudes and SNR (750–8000 Hz)	*Ginkgo biloba* extract was effective in protecting DPOAE amplitude and SNR at 8 kHz
Orgel et al., 2023 [118]	NRCT	n = 23 NAC; n = 24 control	NAC, i.v.	Cisplatin	SIOP ototoxicity scale grade ≥ 2 in the better ear assessed by DPOAE, conventional audiometry (0.5–8 kHz and up to 12.5 kHz), or ABR (0.5–6 kHz)	Receiving NAC following each dose of cisplatin significantly protected hearing at the end of chemotherapy
Yoo et al., 2014 [119]	RCT	n = 11 (one ear treated, other ear control)	NAC, transtympanic	Cisplatin	Auditory thresholds at 2, 4, and 8 kHz assessed by PTA	Transtympanic NAC was not effective
Riga et al., 2013 [120]	NR	n = 20 (one ear treated, other ear control)	NAC, transtympanic	Cisplatin	Auditory thresholds at 250, 500, 1000, 2000, 4000, 8000 Hz assessed by PTA	Transtympanic NAC was effective at 8000 Hz
Sarafraz et al., 2018 [121]	RCT	n = 57 NAC; n = 57 dexamethasone	NAC in one ear and dexamethasone in the other ear, transtympanic	Cisplatin	Auditory thresholds at 250, 500, 1000, 2000, 4000, and 8000 Hz assessed by PTA	Transtympanic NAC was effective at 8000 Hz, dexamethasone was not effective
Yildirim et al., 2010 [122]	RCT	n = 18 NAC, n = 18 salicylate; n = 18 control	NAC or salicylate, i.v.	Cisplatin	High-frequency audiometry (bone and air conduction) at 10 and 12 kHz	NAC was effective in ameliorating bone and air conduction at 10 and 12 kHz; the salicylate effect was not significant
Brock et al., 2018 [107]	RCT	n = 57 sodium thiosulfate; n = 52 control	Sodium thiosulfate 6 h after cisplatin chemotherapy, i.v.	Cisplatin	Auditory thresholds at 1, 2, 4, and 8 kHz were assessed by PTA and categorized according to Brock grades ^§^	Sodium thiosulfate i.v. was effective in preventing hearing loss grade 1 or higher among children with standard-risk hepatoblastoma
Freyer at al., 2017 [106]	RCT	n = 49 sodium thiosulfate; n = 55 control	Sodium thiosulfate 6 h after cisplatin chemotherapy, i.v.	Cisplatin	Hearing loss ^#^ 4 weeks after final cisplatin dose according to auditory thresholds at 0.5–8 kHz determined by PTA or ABR depending on patient status	Sodium thiosulfate i.v. significantly prevented hearing loss in children with various types of cancer
Duinkerken et al., 2021 [123]	RCT	n = 12 (one ear treated, other ear control)	Sodium thiosulfate gel, transtympanic	Cisplatin	Clinically relevant CIHL is defined as ≥ 10 dB auditory thresholds shift average assessed at 8, 10, and 12.5 kHz	Transtympanic sodium thiosulfate showed a non-statistically significant trend towards otoprotection
Rolland et al., 2019 [124]	RCT	n = 13 (one ear treated, other ear control)	Sodium thiosulfate gel, transtympanic	Cisplatin	Permanent auditory threshold shifts (PTSs) assessed by pure tone air conduction (0.5–14 kHz); pure tone bone conduction (0.5–4 kHz); DPOAE (1–6 kHz)	Transtympanic sodium thiosulfate showed a small, non-statistically significant trend towards otoprotection at 3–10 kHz as assessed by pure tone air conduction (PTS)
Viglietta et al., 2020 [125]	RCT	n = 33 sodium thiosulfate; n = 9 control	Sodium thiosulfate pentahydrate formulation, intratympanic	Cisplatin	Safety	Intra-tympanic sodium thiosulfate was safe and well tolerated
Fernandez et al., 2021 [126]	Combined retrospective and prospective observational study	n = 50 atrovastatin; n = 113 any statin; n = 164 control	Statins (various), including atorvastatin, orally	Cisplatin	Incidence of change in hearing based on auditory thresholds shifts as defined by CTCAE ^$^ (1, 2, 3, 4, 6, and 8 kHz) and TUNE (PT average at 1–2-4 and 6–8-12 kHz) criteria [127]	Any statin, and especially atorvastatin, was effective in reducing the incidence of grade 1 or higher hearing loss and auditory threshold shifts at 4, 6, 8, and 12.5 kHz, at any dose
Scasso et al., 2017 [128]	Pilot case–control study	n = 18 supplement; n = 8 control	Supplement consisting of Q10, vitamins B1, B2, B6, B12, E, choline, melatonin, *Ginkgo biloba* extract, and milk protein hydrolysate, orally	Cisplatin	Auditory threshold shifts at 250, 500, 1000, 2000, 4000, and 8000 Hz determined by conventional PTA	Supplement prevented auditory threshold shifts at 8000 Hz in the right ear
Weijl et al., 2004 [129]	RCT	n = 25 supplement; n = 23 control	Supplement consisting of vitamin C, vitamin E, and selenium, orally	Cisplatin	Air or bone conduction threshold at 8 kHz at the start of chemotherapy cycles and 2 months after chemotherapy	No difference was found between the two study arms
Villani et al., 2016 [130]	RCT	n = 13 vitamin E; n = 10 control	Vitamin E, orally	Cisplatin	Auditory threshold shifts at 2000, 4000, and 8000 Hz determined by PTA	Vitamin E was effective in preventing auditory threshold shifts at 2000 and 8000 Hz at 1-month follow-up

ABR, auditory brainstem recording; ASSR, auditory steady-state response; CT, controlled trial; DPOAE, distortion product otoacoustic emission; i.v., intravenous; i.a., intraarterial; NAC, N-acetylcysteine; NR, non-randomized; PTA, pure tone audiometry; RCT, randomized controlled trial; SIOP, International Society of Pediatric Oncology scale; SNR, signal/noise ratio; * assessed as total post-treatment hearing after chemotherapy (the sum of PTA measurements at 6 kHz and 8 kHz in both ears 7 days after the last cisplatin dose), adjusted for baseline total hearing; ^§^ a Brock grade of 0 indicates hearing at less than 40 dB at all the frequencies and does not necessarily corresponds to normal hearing. Grades of 1, 2, 3, and 4 indicate hearing at 40 dB or higher; ^#^ defined as 20 dB or more worsening in pure tone threshold at one test frequency or 10 dB or more worsening at two adjacent test frequencies. ^$^ CTCAE, National Cancer Institute Common Terminology Criteria for Adverse Events (https://ctep.cancer.gov/protocoldevelopment/electronic_applications/docs/ctcae_v5_quick_reference_8.5x11.pdf, accessed on 20 November 2024).

## Data Availability

No new data were created or analyzed in this study. Data sharing is not applicable to this article.

## Supplementary Materials

The following supporting information can be downloaded at https://www.mdpi.com/article/10.3390/antiox13121578/s1, Table S1: Prelinical studies testing antioxidants in cisplatin-induced ototoxicity.
