# Supplementary material for: Cisplatin-Induced Hearing Loss, Oxidative Stress, and Antioxidants as a Therapeutic Strategy—A State-of-the-Art Review
Source: Antioxidants (Basel). 2024 Dec 21;13(12):1578. doi: 10.3390/antiox13121578 (PMC11673797; doi:10.3390/antiox13121578)
Supplement: Supplementary file 1 [file antioxidants-13-01578-s001.zip › antioxidants-3359114-supplementary.pdf]

Table S1: Preclinical studies testing antioxidants in cisplatin-induced ototoxicity with study type, substances and observed effect. PubMed database (https://pubmed.ncbi.nlm.nih.gov/) was searched with the following terms: cisplatin OR oxaliplatin OR carboplatin AND ototoxicity AND antioxidant. Filters: from 2004/7/30 - 2024/7/30.

Study: first author, year	Study type	Tested antioxidant	Platinum-based compound	Otoprotection demonstrated ?
Kim et al., 2017 [1]	Preclinical in vitro	1,2,3-triazole derivative KPR-A020 (1 mM in DMSO)	Cisplatin	Yes
Rybak et al., 2000 [2]	Preclinical in vivo animal	4-methylthiobenzoic acid/diethyldithiocarbamate/ ebselen	Cisplatin	Yes
Zhang et al., 2024 [3]	Preclinical in vivo animal	4-octyl itaconate	Cisplatin	Yes
Li et al., 2023 [4]	Preclinical in vitro	5,7-Dihydroxy-4-methylcoumarin (D4M)	Cisplatin	Yes
Gunes et al., 2011 [5]	Preclinical in vivo animal	Acetyl L-Carnitine	Cisplatin	Yes
Bayrak et al., 2020 [6]	Preclinical in vivo animal	Acetyl L-Carnitine	Cisplatin	Yes
Cai et al., 2019 [7]	Preclinical in vivo animal	Allicin	Cisplatin	Yes
Wu et al., 2017 [8]	Preclinical in vivo animal	Allicin	Cisplatin	Yes
Wu et al., 2017 [9]	Preclinical in vivo animal	Allicin	Cisplatin	Yes
Lynch et al., 2005 [10]	Preclinical in vivo animal	Allopurinol and ebselen	Cisplatin	Yes
Rybak et al., 1999 [11]	Preclinical in vivo animal	Alpha-lipoic acid	Cisplatin	Yes
Kim et al., 2014 [12]	Preclinical in vitro	Alpha-lipoic acid	Cisplatin	Yes
Ozkul et al., 2014 [13]	Preclinical in vivo animal	Alpha-lipoic acid	Cisplatin	Yes
Kim et al., 2018 [14]	Preclinical in vivo animal	Alpha-lipoic acid	Cisplatin	Yes
Curcio et al., 2022 [15]	Preclinical in vitro	Alpha-lipoic acid	Cisplatin	Yes
Cho et al., 2022 [16]	Preclinical in vitro	Alpha-lipoic acid	Cisplatin	Yes

Hyppolito et al., 2005 [17]	Preclinical in vivo animal	Amifostine	Cisplatin	Yes
Cagin et al., 2015 [18]	Preclinical in vivo animal	Apocynin	Cisplatin	Yes
Choi et al., 2013 [19]	Preclinical in vivo animal	Apocynin	Cisplatin	Yes
Kınal et al., 2021 [20]	Preclinical in vivo animal	Astaxanthin	Cisplatin	Yes
Nan et al., 2022 [21]	Preclinical in vivo animal	Astaxanthin	Cisplatin	Yes
Terzi et al., 2021 [22]	Preclinical in vivo animal	Astaxanthin	Cisplatin	Yes
Gu et al., 2020 [23]	Preclinical in vivo animal	Astaxanthin lipid-polymer hybrid nanoparticles (ATX-LPN)	Cisplatin	Yes
Gu et al., 2022 [24]	Preclinical in vivo animal	Astaxanthin ROS-responsive/consuming nanoparticles (ATX-PPS-NP)	Cisplatin	Yes
Xiong et al., 2011 [25]	Preclinical in vivo animal	Astragalosides	Cisplatin	Yes
Kim et al., 2022 [26]	Preclinical in vitro	Berberine chloride	Cisplatin	Yes
Kim et al., 2015 [27]	Preclinical in vivo animal	Bucillamine	Cisplatin	Yes
Kim et al., 2019 [28]	Preclinical in vitro	C -phycocyanin (KNUA002)	Cisplatin	Yes
Kizilay et al., 2004 [29]	Preclinical in vivo animal	Caffeic acid phenethyl ester	Cisplatin	Yes
Choi et al., 2014 [30]	Preclinical in vitro	Caffeic acid, different dosages	Cisplatin	Yes
Khan et al., 2012 [31]	Preclinical in vivo animal	Chrysin	Cisplatin	Yes
Khan et al., 2012 [32]	Preclinical in vivo animal	Chrysin	Cisplatin	Yes
Astolfi et al., 2016 [33]	Preclinical in vivo animal	Coenzyme Q10 plus multivitamin mixture	Cisplatin	Yes
Chen et al., 2024 [34]	Preclinical in vivo animal	Copper chaperon	Cisplatin	Yes

Fetoni et al., 2014 [35]	Preclinical in vivo animal	Curcumin	Cisplatin	Yes
Mendonça et al., 2009 [36]	Preclinical in vitro	Curcumin	Cisplatin	Yes
Salehi et al., 2014 [37]	Preclinical in vivo animal	Curcumin and Dexamethasone	Cisplatin	Yes
Paciello et al., 2020 [38]	Preclinical in vivo animal	Curcumin and ferulic acid	Cisplatin	Yes
Soyalıç et al., 2016 [39]	Preclinical in vivo animal	Curcumin and Vitamin E	Cisplatin	Yes
Hu et al., 2023 [40]	Preclinical in vivo animal	Curcumin loaded graphene oxide quantum dots (CUR/GOQDs)	Cisplatin	Yes
Simsek et al., 2019 [41]	Preclinical in vivo animal	Dexamethasone and resveratol	Cisplatin	Yes
Paksoy et al., 2011 [42]	Preclinical in vivo animal	Dexamethasone and Vitamin E	Cisplatin	Yes
Toplu, et al., 2016 [43]	Preclinical in vivo animal	Dexpanthenol	Cisplatin	Yes
Li et al., 2022 [44]	Preclinical in vivo animal	Dieckol, intratympanic	Cisplatin	Yes
Roldan-Fidalgo, et al., 2014 [45]	Preclinical in vivo animal	Dimethyl sulfoxide (DMSO)	Cisplatin	No
Uribe, et al., 2013 [46]	Preclinical in vivo animal	Dimethyl sulfoxide (DMSO)	Cisplatin	Yes
Campbell et al., 2007 [47]	Preclinical in vivo animal	D-methionine	Cisplatin	Yes
Febles et al., 2022 [48]	Preclinical in vivo animal	D-methionine	Cisplatin	Yes
Giordano et al., 2006 [49]	Preclinical in vivo animal	D-methionine	Cisplatin	Yes
Gopal et al., 2012 [50]	Preclinical in vitro	D-methionine	Cisplatin	Yes
Hinduja et al., 2015 [51]	Preclinical in vivo animal	D-methionine	Cisplatin	Yes
Im et al., 2015 [52]	Preclinical in vitro	Edaravone	Cisplatin	Yes

Hong et al., 2013 [53]	Preclinical in vivo animal	Edaravone	Cisplatin	Yes
Borse et al., 2017 [54]	Preclinical in vivo animal	EGCG (epigallocatechin-3-gallate)	Cisplatin	Yes
Cho et al., 2014 [55]	Preclinical in vitro	EGCG	Cisplatin	Yes
Schmitt et al., 2009 [56]	Preclinical in vivo animal	EGCG	Cisplatin	Yes
Kim et al., 2008 [57]	Preclinical in vitro	Epicatechin	Cisplatin	Yes
Lee at al., 2010 [58]	Preclinical in vivo animal	Epicatechin	Cisplatin	Yes
Kim et al., 2015 [59]	Preclinical in vitro	Erdostein	Cisplatin	Yes
Kalcioglu et al., 2005 [60]	Preclinical in vivo animal	Erdostein	Cisplatin	Yes
Waissbluth et al., 2017 [61]	Preclinical in vivo animal	Erdostein	Cisplatin	Yes
Zhao, et al., 2024 [62]	Preclinical in vivo animal	Ergothioneine	Cisplatin	Yes
Ayral et al., 2021 [63]	Preclinical in vivo animal	Ethyl pyruvate	Cisplatin	Yes
Sakat et al., 2019 [64]	Preclinical in vivo animal	Eugenol	Cisplatin	Yes
Lu et al. 2022 [65]	Preclinical in vivo animal	Eupatilin	Cisplatin	Yes
Hu et al., 2020 [66]	Preclinical in vitro	Ferrostatin-1	Cisplatin	Yes
Jo et al., 2019 [67]	Preclinical in vitro	Ferulic acid	Cisplatin	Yes
Li et al., 2024 [68]	Preclinical in vitro	Formononetin	Cisplatin	Yes
Guo et al., 2018 [69]	Preclinical in vitro	Forskolin	Cisplatin	Yes
Hsieh et al., 2023 [70]	Preclinical in vitro	Fucoidan	Cisplatin	Yes
Kim et al., 2021 [71]	Preclinical in vitro	Fursultiamine in DMSO	Cisplatin	Yes
Tan et al., 2022 [72]	Preclinical in vivo animal	Genistein	Cisplatin	Yes

Lo et al., 2017 [73]	Preclinical in vivo animal	Geranylgeranylacetone	Cisplatin	Yes
Choi et al., 2013 [74]	Preclinical in vivo animal	*Gingko biloba* extract	Cisplatin	Yes
Esen et al., 2018 [75]	Preclinical in vivo animal	*Gingko biloba* extract and lycopene	Cisplatin	Yes
Huang et al., 2007 [76]	Preclinical in vivo animal	*Gingko biloba* extract	Cisplatin	Yes
Xu et al., 2004 [77]	Preclinical in vivo animal	*Ginkgo* leaf and Deferoxamine	Cisplatin	Yes
Fei et al., 2020 [78]	Preclinical in vitro	Ginseng	Cisplatin	Yes
Im et al., 2010 [79]	Preclinical in vitro	Ginseng	Cisplatin	Yes
Qiao et al., 2023 [80]	Preclinical in vitro	Ginseng	Cisplatin	Yes
Lou et al., 2024 [81]	Preclinical in vivo animal	Hesperidin	Cisplatin	Yes
Kara et al., 2016 [82]	Preclinical in vivo animal	Hesperetin	Cisplatin	Yes
Kikkawa et al., 2014 [83]	Preclinical in vivo animal	Hydrogen	Cisplatin	Yes
Qu et al., 2012 [84]	Preclinical in vivo animal	Hydrogen	Cisplatin	Yes
Zhang et al., 2020 [85]	Preclinical in vitro	Hydroxytyrosol	Cisplatin	No
Bekmez Bilmez et al., 2016 [86]	Preclinical in vivo animal	Intratympanic and intraperitoneal oxytocin	Cisplatin	Yes
Celebi et al., 2013 [87]	Preclinical in vivo animal	Intratympanic injection of vitamin C	Cisplatin	Yes
Wang et al. 2024 [88]	Preclinical in vivo animal	Isoselenazolones	Cisplatin	Yes
Alan et al., 2018 [89]	Preclinical in vivo animal	Isotretinoin	Cisplatin	Yes
Lee et al., 2020 [90]	Preclinical in vitro	KL 1333 (derivative of β-lapachone)	Cisplatin	Yes
Nader et al., 2010 [91]	Preclinical in vivo animal	Lactate and N-acetylcysteine	Cisplatin	Yes

Abd-Allah et al., 2005 [92]	Preclinical in vitro	L-Carnitine	Carboplatin	Yes
Gozeler et al., 2019 [93]	Preclinical in vivo animal	Levosimendan	Cisplatin	Yes
Koo et al., 2016 [94]	Preclinical in vitro	Lipoic acid and glutathione	Cisplatin	Yes
Li et al., 2015 [95]	Preclinical in vitro	LSD (lysine-specific demethylase) 1 inhibitors	Cisplatin	Yes
Roldàn-Fidalgo et al., 2016 [96]	Preclinical in vivo animal	Lutein	Cisplatin	In vitro: yes, in vivo: no
Ciçek et al., 2014 [97]	Preclinical in vivo animal	Lycopene	Cisplatin	Yes
Ozkırıs et al. 2013 [98]	Preclinical in vivo animal	Lycopene	Cisplatin	Yes
Noman et al., 2022 [99]	Preclinical in vivo animal	Mannitol	Cisplatin	Yes
Kasse et al., 2008 [100]	Preclinical in vivo animal	*Maytenus ilicifolia*	Cisplatin	Yes
Araujo et al., 2019 [101]	Preclinical in vivo animal	Melatonin	Cisplatin	Yes
Assumpção et al., 2023 [102]	Preclinical in vivo animal	Melatonin	Cisplatin	Yes
Demir et al., 2015 [103]	Preclinical in vivo animal	Melatonin	Cisplatin	Yes
Cappaert et al., 2005 [104]	Preclinical in vivo animal	Methylthiobenzoic acid and natriumthiosulfat	Cisplatin	Yes
Doğan et al., 2016 [105]	Preclinical in vivo animal	Misoprostol	Cisplatin	Yes
Tate et al., 2017 [106]	Preclinical in vivo animal	Mitoquinone	Cisplatin	Yes
Bentli et al., 2013 [107]	Preclinical in vivo animal	Molsidomine	Cisplatin	Yes
Toplu et al., 2014 [108]	Preclinical in vivo animal	Molsidomine	Cisplatin	Yes
Chen et al., 2022 [109]	Preclinical in vivo animal	N-acetylcysteine	Cisplatin	Yes

Dickey et al., 2005 [110]	Preclinical in vivo animal	N-acetylcysteine	Cisplatin	Yes
Kedeshian et al., 2023 [111]	Preclinical in vitro	N-acetylcysteine	Cisplatin	Yes
Muldoon et al., 2015 [112]	Preclinical in vivo animal	N-acetylcysteine	Cisplatin	Yes
Okur et al., 2007 [113]	Preclinical in vivo animal	N-acetylcysteine	Carboplatin	Yes
Somdaş et al., 2020 [114]	Preclinical in vivo animal	N-acetylcysteine	Cisplatin	Yes
Wang et al., 2022 [115]	Preclinical in vivo animal	N-acetylcysteine	Cisplatin	Yes
Wu et al., 2005 [116]	Preclinical in vitro	N-acetylcysteine	Cisplatin	Yes
Moon et al., 2011 [117]	Preclinical in vitro	N-acetylcysteine	Carboplatin	Yes
Choe et al., 2004 [118]	Preclinical in vivo animal	N-acetylcysteine, ringer lactate	Cisplatin	Yes
Sooriyaarachchi et al., 2013 [119]	Preclinical in vitro	N-acetylcysteine	Cisplatin	Yes
Akaras et al., 2024 [120]	Preclinical in vivo animal	Naringin	Oxaliplatin	Yes
Li et al., 2021 [121]	Preclinical in vivo animal	Naringin	Cisplatin	Yes
Dailan et al., 2013 [122]	Preclinical in vitro	Nicotinamide adenine dinucleotide (NAD+)	Oxaliplatin	Yes
Song et al., 2024 [123]	Preclinical in vitro	Nobiletin	Cisplatin	Yes
Gao et al., 2024 [124]	Preclinical in vivo animal	Nuciferine	Cisplatin	Yes
Youn et al., 2017 [125]	Preclinical in vitro	Peanut sprout extract	Cisplatin	Yes
Li et al., 2022 [126]	Preclinical in vivo animal	Polydatin	Cisplatin	Yes
Yazici et al., 2012 [127]	Preclinical in vivo animal	Pomegranate extract	Cisplatin	Yes
Xu et al., 2022 [128]	Preclinical in vitro	Puerarin	Cisplatin	Yes

Gündoğdu et al., 2019 [129]	Preclinical in vivo animal	Quercetin	Cisplatin	Yes
Huang et al., 2024 [130]	Preclinical in vivo animal	Quercetin	Cisplatin	Yes
Lee et al., 2015 [131]	Preclinical in vivo animal	Quercetin	Cisplatin	Yes
Najafi et al. 2022 [132]	Preclinical in vivo animal	Quercetin	Cisplatin	Yes
Alsaikhan et al., 2023 [133]	Preclinical in vivo animal	Resveratrol	Cisplatin	Yes
Erdem et al., 2012 [134]	Preclinical in vivo animal	Resveratrol	Cisplatin	Yes
Lee et al., 2020 [135]	Preclinical in vivo animal	Resveratrol	Cisplatin	Yes
Lee et al., 2015 [136]	Preclinical in vitro	Resveratrol	Cisplatin	Yes
Liu et al., 2021 [137]	Preclinical in vitro	Resveratrol	Cisplatin	Yes
Olgun et al., 2014 [138]	Preclinical in vivo animal	Resveratrol	Cisplatin	Yes
Simsek et al., 2013 [139]	Preclinical in vivo animal	Resveratrol	Cisplatin	Yes
Yumusakhuylu et al., 2012 [140]	Preclinical in vivo animal	Resveratrol	Cisplatin	Yes
Ustun Bezgin et al., 2019 [141]	Preclinical in vivo animal	Riluzole	Cisplatin	Yes
Munguia et al., 2010 [142]	Preclinical in vivo animal	Ringer's lactate	Cisplatin	No
Zheng et al., 2020 [143]	Preclinical in vitro	Salvianolic acid B	Cisplatin	Yes
Li et al., 2024 [144]	Preclinical in vivo animal	Schisandrin B	Cisplatin	Yes
Doğan et al., 2016 [145]	Preclinical in vivo animal	Selenium	Cisplatin	Yes
Cho et al., 2014 [146]	Preclinical in vitro	Silymarin	Cisplatin	Yes

Hyppolito et al., 2006 [147]	Preclinical in vivo animal	Sodium salicylate	Cisplatin	Yes
Pierre et al., 2009 [148]	Preclinical in vivo animal	Sodium thiosulfate	Cisplatin	Yes
Youn et al., 2016 [149]	Preclinical in vitro	Tempol	Cisplatin	Yes
Kuduban et al., 2013 [150]	Preclinical in vivo animal	Thiamine Pyrophosphate (TPP)	Cisplatin	Yes
Sagit et al., 2013 [151]	Preclinical in vivo animal	Thymoquinone	Cisplatin	Yes
Di et al., 2020 [152]	Preclinical in vivo animal	Ursolic acid	Cisplatin	Yes
Kim et al., 2016 [153]	Preclinical in vitro	Vitamin E	Cisplatin	Yes
Özdemir et al., 2019 [154]	Preclinical in vivo animal	Whortleberry extract	Cisplatin	Yes
Lee et al., 2020 [155]	Preclinical in vivo animal	Zingerone	Cisplatin	Yes

References

1. Kim, Y.-R.; Da Jung, J.; Oh, S.-K.; Lee, T.; Lee, I.-K.; Lee, K.-Y.; Kim, U.-K. Protective effects of 1,2,3-triazole derivative KPR-A020 against cisplatin-induced ototoxicity in murine cochlear cultures. *Int. J. Pediatr. Otorhinolaryngol.* **2017**, *96*, 59–64, doi:10.1016/j.ijporl.2017.02.028.
2. Rybak, L.P.; Husain, K.; Morris, C.; Whitworth, C.; Somani, S. Effect of protective agents against cisplatin ototoxicity. *Am. J. Otol.* **2000**, *21*, 513–520.
3. Zhang, L.; Song, W.; Li, H.; Cui, X.; Ma, J.; Wang, R.; Xu, Y.; Li, M.; Bai, X.; Wang, D.; et al. 4-octyl itaconate alleviates cisplatin-induced ferroptosis possibly via activating the NRF2/HO-1 signalling pathway. *J. Cell. Mol. Med.* **2024**, *28*, e18207, doi:10.1111/jcmm.18207.
4. Li, C.; Wang, X.; Qiao, X.; Fan, L.; Zhu, H.; Chen, Y.; He, Y.; Zhang, Z. 5,7-Dihydroxy-4-methylcoumarin modulates the JNK/FoxO1 signaling pathway to attenuate cisplatin-induced ototoxicity by suppressing oxidative stress and apoptosis in vitro. *Biochim. Biophys. Acta Mol. Cell Res.* **2023**, *1870*, 119437, doi:10.1016/j.bbamcr.2023.119437.
5. Gunes, D.; Kirkim, G.; Kolatan, E.; Guneri, E.A.; Ozogul, C.; Altun, Z.; Serbetcioglu, B.; Yilmaz, O.; Aktas, S.; Mutafoglu, K.; et al. Evaluation of the effect of acetyl L-carnitine on experimental cisplatin ototoxicity and neurotoxicity. *Chemotherapy* **2011**, *57*, 186–194, doi:10.1159/000323621.
6. Bayrak, S.; Aktaş, S.; Altun, Z.; Çakir, Y.; Tütüncü, M.; Kum Özşengezer, S.; Yilmaz, O.; Olgun, N. Antioxidant effect of acetyl-l-carnitine against cisplatin-induced cardiotoxicity. *J. Int. Med. Res.* **2020**, *48*, 300060520951393, doi:10.1177/0300060520951393.
7. Cai, J.; Wu, X.; Li, X.; Ma, C.; Xu, L.; Guo, X.; Li, J.; Wang, H.; Han, Y. Allicin Protects against Cisplatin-Induced Stria Vascularis Damage: Possible Relation to Inhibition of Caspase-3 and

PARP-1-AIF-Mediated Apoptotic Pathways. *ORL J. Otorhinolaryngol. Relat. Spec.* **2019**, *81*, 202–214, doi:10.1159/000500557.
8. Wu, X.; Cai, J.; Li, X.; Li, H.; Li, J.; Bai, X.; Liu, W.; Han, Y.; Xu, L.; Zhang, D.; et al. Allicin protects against cisplatin-induced vestibular dysfunction by inhibiting the apoptotic pathway. *Eur. J. Pharmacol.* **2017**, *805*, 108–117, doi:10.1016/j.ejphar.2017.02.052.
9. Wu, X.; Li, X.; Song, Y.; Li, H.; Bai, X.; Liu, W.; Han, Y.; Xu, L.; Li, J.; Zhang, D.; et al. Allicin protects auditory hair cells and spiral ganglion neurons from cisplatin - Induced apoptosis. *Neuropharmacology* **2017**, *116*, 429–440, doi:10.1016/j.neuropharm.2017.01.001.
10. Lynch, E.D.; Gu, R.; Pierce, C.; Kil, J. Reduction of acute cisplatin ototoxicity and nephrotoxicity in rats by oral administration of allopurinol and ebselen. *Hear. Res.* **2005**, *201*, 81–89, doi:10.1016/j.heares.2004.08.002.
11. Rybak, L.P.; Husain, K.; Whitworth, C.; Somani, S.M. Dose dependent protection by lipoic acid against cisplatin-induced ototoxicity in rats: antioxidant defense system. *Toxicol. Sci.* **1999**, *47*, 195–202, doi:10.1093/toxsci/47.2.195.
12. Kim, J.; Cho, H.-J.; Sagong, B.; Kim, S.-J.; Lee, J.-T.; So, H.-S.; Lee, I.-K.; Kim, U.-K.; Lee, K.-Y.; Choo, Y.-S. Alpha-lipoic acid protects against cisplatin-induced ototoxicity via the regulation of MAPKs and proinflammatory cytokines. *Biochem. Biophys. Res. Commun.* **2014**, *449*, 183–189, doi:10.1016/j.bbrc.2014.04.118.
13. Ozkul, Y.; Songu, M.; Basoglu, M.S.; Ozturkcan, S.; Katilmis, H. Evaluation of the protective effect of α-lipoic acid on cisplatin ototoxicity using distortion-product otoacoustic emission measurements: an experimental animal study. *J. Craniofac. Surg.* **2014**, *25*, 1515–1518, doi:10.1097/SCS.0000000000000881.
14. Kim, K.-H.; Lee, B.; Kim, Y.-R.; Kim, M.-A.; Ryu, N.; Da Jung, J.; Kim, U.-K.; Baek, J.-I.; Lee, K.-Y. Evaluating protective and therapeutic effects of alpha-lipoic acid on cisplatin-induced ototoxicity. *Cell Death Dis.* **2018**, *9*, 827, doi:10.1038/s41419-018-0888-z.
15. Curcio, M.; Cirillo, G.; Amato, R.; Guidotti, L.; Amantea, D.; Luca, M. de; Nicoletta, F.P.; Iemma, F.; Garcia-Gil, M. Encapsulation of Alpha-Lipoic Acid in Functional Hybrid Liposomes: Promising Tool for the Reduction of Cisplatin-Induced Ototoxicity. *Pharmaceuticals (Basel)* **2022**, *15*, doi:10.3390/ph15040394.
16. Cho, S.; Hong, S.J.; Kang, S.H.; Park, Y.; Kim, S.K. Alpha-Lipoic Acid Attenuates Apoptosis and Ferroptosis in Cisplatin-Induced Ototoxicity via the Reduction of Intracellular Lipid Droplets. *Int. J. Mol. Sci.* **2022**, *23*, doi:10.3390/ijms231810981.
17. Hyppolito, M.A.; Oliveira, A.A. de; Lessa, R.M.; Rossato, M. Amifostine otoprotection to cisplatin ototoxicity: a guinea pig study using otoacoustic emission distortion products (DPOEA) and scanning electron microscopy. *Braz. J. Otorhinolaryngol.* **2005**, *71*, 268–273, doi:10.1016/s1808-8694(15)31322-7.
18. Cagin, Y.F.; Erdogan, M.A.; Sahin, N.; Parlakpinar, H.; Atayan, Y.; Polat, A.; Vardi, N.; Yildiz, A.; Tanbek, K. Protective Effects of Apocynin on Cisplatin-induced Hepatotoxicity in Rats. *Arch. Med. Res.* **2015**, *46*, 517–526, doi:10.1016/j.arcmed.2015.08.005.
19. Choi, J.; Im, G.J.; Chang, J.; Chae, S.W.; Lee, S.H.; Kwon, S.-Y.; Chung, A.-Y.; Park, H.-C.; Jung, H.H. Protective effects of apocynin on cisplatin-induced ototoxicity in an auditory cell line and in zebrafish. *J. Appl. Toxicol.* **2013**, *33*, 125–133, doi:10.1002/jat.1729.
20. Kınal, M.E.; Tatlıpınar, A.; Uzun, S.; Keskin, S.; Tekdemir, E.; Özbeyli, D.; Akakın, D. Investigation of Astaxanthin Effect on Cisplatin Ototoxicity in Rats by Using Otoacoustic Emission, Total Antioxidant Capacity, and Histopathological Methods. *Ear Nose Throat J.* **2021**, *100*, NP198-NP205, doi:10.1177/0145561319866826.
21. Nan, B.; Zhao, Z.; Jiang, K.; Gu, X.; Li, H.; Huang, X. Astaxanthine attenuates cisplatin ototoxicity in vitro and protects against cisplatin-induced hearing loss in vivo. *Acta Pharm. Sin. B* **2022**, *12*, 167–181, doi:10.1016/j.apsb.2021.07.002.

22. Terzi, S.; Özgür, A.; Çeliker, M.; Mercantepe, T.; Yilmaz, A.; Tümkaya, L.; Kaya, Ş.; Demir, E.; Dursun, E. The protective effect of astaxanthin on cisplatin-induced ototoxicity. *Adv. Clin. Exp. Med.* **2021**, *30*, 315–321, doi:10.17219/acem/133081.
23. Gu, J.; Chen, Y.; Tong, L.; Wang, X.; Yu, D.; Wu, H. Astaxanthin-loaded polymer-lipid hybrid nanoparticles (ATX-LPN): assessment of potential otoprotective effects. *J. Nanobiotechnology* **2020**, *18*, 53, doi:10.1186/s12951-020-00600-x.
24. Gu, J.; Wang, X.; Chen, Y.; Xu, K.; Yu, D.; Wu, H. An enhanced antioxidant strategy of astaxanthin encapsulated in ROS-responsive nanoparticles for combating cisplatin-induced ototoxicity. *J. Nanobiotechnology* **2022**, *20*, 268, doi:10.1186/s12951-022-01485-8.
25. Xiong, M.; He, Q.; Wang, J.; Lai, H. Astragalosides reduce cisplatin ototoxicity in guinea pigs. *ORL J. Otorhinolaryngol. Relat. Spec.* **2011**, *73*, 131–136, doi:10.1159/000326240.
26. Kim, J.-H.; Baek, J.-I.; Lee, I.-K.; Kim, U.-K.; Kim, Y.-R.; Lee, K.-Y. Protective effect of berberine chloride against cisplatin-induced ototoxicity. *Genes Genomics* **2022**, *44*, 1–7, doi:10.1007/s13258-021-01157-1.
27. Kim, S.-J.; Ho Hur, J.; Park, C.; Kim, H.-J.; Oh, G.-S.; Lee, J.N.; Yoo, S.-J.; Choe, S.-K.; So, H.-S.; Lim, D.J.; et al. Bucillamine prevents cisplatin-induced ototoxicity through induction of glutathione and antioxidant genes. *Exp. Mol. Med.* **2015**, *47*, e142, doi:10.1038/emm.2014.112.
28. Kim, Y.-R.; Do, J.-M.; Kim, K.H.; Stoica, A.R.; Jo, S.-W.; Kim, U.-K.; Yoon, H.-S. C-phycocyanin from Limnothrix Species KNUA002 Alleviates Cisplatin-Induced Ototoxicity by Blocking the Mitochondrial Apoptotic Pathway in Auditory Cells. *Mar. Drugs* **2019**, *17*, doi:10.3390/md17040235.
29. Kizilay, A.; Kalcioglu, M.T.; Ozerol, E.; Iraz, M.; Gulec, M.; Akyol, O.; Ozturan, O. Caffeic acid phenethyl ester ameliorated ototoxicity induced by cisplatin in rats. *J. Chemother.* **2004**, *16*, 381–387, doi:10.1179/joc.2004.16.4.381.
30. Choi, J.; Kim, S.H.; Rah, Y.C.; Chae, S.W.; Lee, J.D.; Lee, B.D.; Park, M.K. Effects of caffeic acid on cisplatin-induced hair cell damage in HEI-OC1 auditory cells. *Int. J. Pediatr. Otorhinolaryngol.* **2014**, *78*, 2198–2204, doi:10.1016/j.ijporl.2014.10.013.
31. Khan, R.; Khan, A.Q.; Qamar, W.; Lateef, A.; Ali, F.; Rehman, M.U.; Tahir, M.; Sharma, S.; Sultana, S. Chrysin abrogates cisplatin-induced oxidative stress, p53 expression, goblet cell disintegration and apoptotic responses in the jejunum of Wistar rats. *Br. J. Nutr.* **2012**, *108*, 1574–1585, doi:10.1017/S0007114511007239.
32. Khan, R.; Khan, A.Q.; Qamar, W.; Lateef, A.; Tahir, M.; Rehman, M.U.; Ali, F.; Sultana, S. Chrysin protects against cisplatin-induced colon. toxicity via amelioration of oxidative stress and apoptosis: probable role of p38MAPK and p53. *Toxicol. Appl. Pharmacol.* **2012**, *258*, 315–329, doi:10.1016/j.taap.2011.11.013.
33. Astolfi, L.; Simoni, E.; Valente, F.; Ghiselli, S.; Hatzopoulos, S.; Chicca, M.; Martini, A. Coenzyme Q10 plus Multivitamin Treatment Prevents Cisplatin Ototoxicity in Rats. *PLoS One* **2016**, *11*, e0162106, doi:10.1371/journal.pone.0162106.
34. Chen, X.; Xiang, W.; Li, L.; Xu, K. Copper Chaperone Atox1 Protected the Cochlea From Cisplatin by Regulating the Copper Transport Family and Cell Cycle. *Int. J. Toxicol.* **2024**, *43*, 134–145, doi:10.1177/10915818231206665.
35. Fetoni, A.R.; Eramo, S.L.M.; Paciello, F.; Rolesi, R.; Podda, M.V.; Troiani, D.; Paludetti, G. Curcuma longa (curcumin) decreases in vivo cisplatin-induced ototoxicity through heme oxygenase-1 induction. *Otol. Neurotol.* **2014**, *35*, e169-77, doi:10.1097/MAO.0000000000000302.
36. Mendonça, L.M.; Dos Santos, G.C.; Antonucci, G.A.; Dos Santos, A.C.; Bianchi, Maria de Lourdes Pires; Antunes, L.M.G. Evaluation of the cytotoxicity and genotoxicity of curcumin in PC12 cells. *Mutat. Res.* **2009**, *675*, 29–34, doi:10.1016/j.mrgentox.2009.02.003.
37. Salehi, P.; Akinpelu, O.V.; Waissbluth, S.; Peleva, E.; Meehan, B.; Rak, J.; Daniel, S.J. Attenuation of cisplatin ototoxicity by otoprotective effects of nanoencapsulated curcumin and dexamethasone

in a guinea pig model. *Otol. Neurotol.* **2014**, *35*, 1131–1139, doi:10.1097/MAO.0000000000000403.
38. Paciello, F.; Fetoni, A.R.; Mezzogori, D.; Rolesi, R.; Di Pino, A.; Paludetti, G.; Grassi, C.; Troiani, D. The dual role of curcumin and ferulic acid in counteracting chemoresistance and cisplatin-induced ototoxicity. *Sci. Rep.* **2020**, *10*, 1063, doi:10.1038/s41598-020-57965-0.
39. Soyalıç, H.; Gevrek, F.; Koç, S.; Avcu, M.; Metin, M.; Aladağ, İ. Intraperitoneal curcumin and vitamin E combination for the treatment of cisplatin-induced ototoxicity in rats. *Int. J. Pediatr. Otorhinolaryngol.* **2016**, *89*, 173–178, doi:10.1016/j.ijporl.2016.08.012.
40. an Hu; Zhang, J.-W.; Yang, L.-Y.; Qiao, P.-P.; Lu, D.; Yu, Y.-F. Curcumin-loaded graphene oxide quantum dots enhance otoprotective effects via blocking cuproptosis. *Front. Bioeng. Biotechnol.* **2023**, *11*, 1183197, doi:10.3389/fbioe.2023.1183197.
41. Simsek, G.; Taş, B.M.; Muluk, N.B.; Azman, M.; Kılıç, R. Comparison of the protective efficacy between intratympanic dexamethasone and resveratrol treatments against cisplatin-induced ototoxicity: an experimental study. *Eur. Arch. Otorhinolaryngol.* **2019**, *276*, 3287–3293, doi:10.1007/s00405-019-05635-x.
42. Paksoy, M.; Ayduran, E.; Sanlı, A.; Eken, M.; Aydın, S.; Oktay, Z.A. The protective effects of intratympanic dexamethasone and vitamin E on cisplatin-induced ototoxicity are demonstrated in rats. *Med. Oncol.* **2011**, *28*, 615–621, doi:10.1007/s12032-010-9477-4.
43. Toplu, Y.; Sapmaz, E.; Parlakpinar, H.; Kelles, M.; Kalcioglu, M.T.; Tanbek, K.; Kizilay, A. The Effect of Dexpanthenol on Ototoxicity Induced by Cisplatin. *Clin. Exp. Otorhinolaryngol.* **2016**, *9*, 14–20, doi:10.21053/ceo.2016.9.1.14.
44. Li, H.; Oh, S.H.; Shin, H.-C.; Suh, M.-W. Intratympanic Administration of Dieckol Prevents Ototoxic Hearing Loss. *Mar. Drugs* **2022**, *20*, doi:10.3390/md20100622.
45. Roldán-Fidalgo, A.; Trinidad, A.; Rodríguez-Valiente, A.; García-Berrocal, J.R.; Millán, I.; Coronado, M.J.; Ramírez-Camacho, R. Effect of intratympanic dimethyl sulphoxide (DMSO) in an in vivo model of cisplatin-related ototoxicity. *Eur. Arch. Otorhinolaryngol.* **2014**, *271*, 3121–3126, doi:10.1007/s00405-014-2957-y.
46. Uribe, P.M.; Mueller, M.A.; Gleichman, J.S.; Kramer, M.D.; Wang, Q.; Sibrian-Vazquez, M.; Strongin, R.M.; Steyger, P.S.; Cotanche, D.A.; Matsui, J.I. Dimethyl sulfoxide (DMSO) exacerbates cisplatin-induced sensory hair cell death in zebrafish (Danio rerio). *PLoS One* **2013**, *8*, e55359, doi:10.1371/journal.pone.0055359.
47. Campbell, K.C.M.; Meech, R.P.; Klemens, J.J.; Gerberi, M.T.; Dyrstad, S.S.W.; Larsen, D.L.; Mitchell, D.L.; El-Azizi, M.; Verhulst, S.J.; Hughes, L.F. Prevention of noise- and drug-induced hearing loss with D-methionine. *Hear. Res.* **2007**, *226*, 92–103, doi:10.1016/j.heares.2006.11.012.
48. Febles, N.K.; Bauer, M.A.; Ding, B.; Zhu, X.; Gallant, N.D.; Frisina, R.D. A combinatorial approach to protect sensory tissue against cisplatin-induced ototoxicity. *Hear. Res.* **2022**, *415*, 108430, doi:10.1016/j.heares.2022.108430.
49. Giordano, P.; Lorito, G.; Ciorba, A.; Martini, A.; Hatzopoulos, S. Protection against cisplatin ototoxicity in a Sprague-Dawley rat animal model. *Acta Otorhinolaryngol. Ital.* **2006**, *26*, 198–207.
50. Gopal, K.V.; Wu, C.; Shrestha, B.; Campbell, K.C.M.; Moore, E.J.; Gross, G.W. d-Methionine protects against cisplatin-induced neurotoxicity in cortical networks. *Neurotoxicol. Teratol.* **2012**, *34*, 495–504, doi:10.1016/j.ntt.2012.06.002.
51. Hinduja, S.; Kraus, K.S.; Manohar, S.; Salvi, R.J. D-methionine protects against cisplatin-induced neurotoxicity in the hippocampus of the adult rat. *Neurotox. Res.* **2015**, *27*, 199–204, doi:10.1007/s12640-014-9503-y.
52. Im, G.J.; Chang, J.; Lee, S.; Choi, J.; Jung, H.H.; Lee, H.M.; Ryu, S.H.; Park, S.K.; Kim, J.H.; Kim, H.-J. Protective role of edaravone against cisplatin-induced ototoxicity in an auditory cell line. *Hear. Res.* **2015**, *330*, 113–118, doi:10.1016/j.heares.2015.08.004.

53. Hong, S.J.; Im, G.J.; Chang, J.; Chae, S.W.; Lee, S.H.; Kwon, S.Y.; Jung, H.H.; Chung, A.Y.; Park, H.C.; Choi, J. Protective effects of edaravone against cisplatin-induced hair cell damage in zebrafish. *Int. J. Pediatr. Otorhinolaryngol.* **2013**, *77*, 1025–1031, doi:10.1016/j.ijporl.2013.04.003.
54. Borse, V.; Al Aameri, Raheem F H; Sheehan, K.; Sheth, S.; Kaur, T.; Mukherjea, D.; Tupal, S.; Lowy, M.; Ghosh, S.; Dhukhwa, A.; et al. Epigallocatechin-3-gallate, a prototypic chemopreventative agent for protection against cisplatin-based ototoxicity. *Cell Death Dis.* **2017**, *8*, e2921, doi:10.1038/cddis.2017.314.
55. Cho, S.I.; Lee, J.H.; Park, J.H.; Do, N.Y. Protective effect of (-)-epigallocatechin-3-gallate against cisplatin-induced ototoxicity. *J. Laryngol. Otol.* **2014**, 1–6, doi:10.1017/S0022215114000553.
56. Schmitt, N.C.; Rubel, E.W.; Nathanson, N.M. Cisplatin-induced hair cell death requires STAT1 and is attenuated by epigallocatechin gallate. *J. Neurosci.* **2009**, *29*, 3843–3851, doi:10.1523/JNEUROSCI.5842-08.2009.
57. Kim, C.-H.; Kang, S.U.; Pyun, J.; Lee, M.H.; Hwang, H.S.; Lee, H. Epicatechin protects auditory cells against cisplatin-induced death. *Apoptosis* **2008**, *13*, 1184–1194, doi:10.1007/s10495-008-0242-5.
58. Lee, J.S.; Kang, S.U.; Hwang, H.S.; Pyun, J.H.; Choung, Y.H.; Kim, C.H. Epicatechin protects the auditory organ by attenuating cisplatin-induced ototoxicity through inhibition of ERK. *Toxicol. Lett.* **2010**, *199*, 308–316, doi:10.1016/j.toxlet.2010.09.013.
59. Kim, S.-J.; Park, C.; Lee, J.N.; Lim, H.; Hong, G.-Y.; Moon, S.K.; Lim, D.J.; Choe, S.-K.; Park, R. Erdosteine protects HEI-OC1 auditory cells from cisplatin toxicity through suppression of inflammatory cytokines and induction of Nrf2 target proteins. *Toxicol. Appl. Pharmacol.* **2015**, *288*, 192–202, doi:10.1016/j.taap.2015.07.014.
60. Kalcioglu, M.T.; Kizilay, A.; Gulec, M.; Karatas, E.; Iraz, M.; Akyol, O.; Egri, M.; Ozturan, O. The protective effect of erdosteine against ototoxicity induced by cisplatin in rats. *Eur. Arch. Otorhinolaryngol.* **2005**, *262*, 856–863, doi:10.1007/s00405-004-0909-7.
61. Waissbluth, S.; Garnier, D.; Akinpelu, O.V.; Salehi, P.; Daniel, S.J. The impact of erdosteine on cisplatin-induced ototoxicity: a proteomics approach. *Eur. Arch. Otorhinolaryngol.* **2017**, *274*, 1365–1374, doi:10.1007/s00405-016-4399-1.
62. Zhao, W.; Wu, F.; Hu, R.; Lou, J.; Chen, G.; Cai, Z.; Chen, S. The Antioxidant Ergothioneine Alleviates Cisplatin-Induced Hearing Loss through the Nrf2 Pathway. *Antioxid. Redox Signal.* **2024**, doi:10.1089/ars.2024.0648.
63. Ayral, M.; Toprak, S.F. The effects of ethyl pyruvate against experimentally induced cisplatin ototoxicity in rats. *Somatosens. Mot. Res.* **2021**, *38*, 347–352, doi:10.1080/08990220.2021.1984875.
64. Sakat, M.S.; Kilic, K.; Akdemir, F.N.E.; Yildirim, S.; Eser, G.; Kiziltunc, A. The effectiveness of eugenol against cisplatin-induced ototoxicity. *Braz. J. Otorhinolaryngol.* **2019**, *85*, 766–773, doi:10.1016/j.bjorl.2018.07.007.
65. Lu, X.; Deng, T.; Dong, H.; Han, J.; Yu, Y.; Xiang, D.; Nie, G.; Hu, B. Novel Application of Eupatilin for Effectively Attenuating Cisplatin-Induced Auditory Hair Cell Death via Mitochondrial Apoptosis Pathway. *Oxid. Med. Cell. Longev.* **2022**, *2022*, 1090034, doi:10.1155/2022/1090034.
66. Hu, B.; Liu, Y.; Chen, X.; Zhao, J.; Han, J.; Dong, H.; Zheng, Q.; Nie, G. Ferrostatin-1 protects auditory hair cells from cisplatin-induced ototoxicity in vitro and in vivo. *Biochem. Biophys. Res. Commun.* **2020**, *533*, 1442–1448, doi:10.1016/j.bbrc.2020.10.019.
67. Jo, E.-R.; Youn, C.K.; Jun, Y.; Cho, S.I. The protective role of ferulic acid against cisplatin-induced ototoxicity. *Int. J. Pediatr. Otorhinolaryngol.* **2019**, *120*, 30–35, doi:10.1016/j.ijporl.2019.02.001.
68. Li, Y.; Wu, J.; Yu, H.; Lu, X.; Ni, Y. Formononetin ameliorates cisplatin-induced hair cell death via activation of the PI3K/AKT-Nrf2 signaling pathway. *Heliyon* **2024**, *10*, e23750, doi:10.1016/j.heliyon.2023.e23750.

69. Guo, X.; Bai, X.; Li, L.; Li, J.; Wang, H. Forskolin protects against cisplatin-induced ototoxicity by inhibiting apoptosis and ROS production. *Biomed. Pharmacother.* **2018**, *99*, 530–536, doi:10.1016/j.biopha.2018.01.080.
70. Hsieh, C.-Y.; Lin, J.-N.; Kang, T.-Y.; Wen, Y.-H.; Yu, S.-H.; Wu, C.-C.; Wu, H.-P. Otoprotective Effects of Fucoidan Reduce Cisplatin-Induced Ototoxicity in Mouse Cochlear UB/OC-2 Cells. *Int. J. Mol. Sci.* **2023**, *24*, doi:10.3390/ijms24043561.
71. Kim, Y.-R.; Kwon, T.-J.; Kim, U.-K.; Lee, I.-K.; Lee, K.-Y.; Baek, J.-I. Fursultiamine Prevents Drug-Induced Ototoxicity by Reducing Accumulation of Reactive Oxygen Species in Mouse Cochlea. *Antioxidants (Basel)* **2021**, *10*, doi:10.3390/antiox10101526.
72. Tan, M.; Toplu, Y.; Varan, E.; Sapmaz, E.; Özhan, O.; Parlakpınar, H.; Polat, A. The effect of genistein on cisplatin induced ototoxicity and oxidative stress. *Braz. J. Otorhinolaryngol.* **2022**, *88*, 105–111, doi:10.1016/j.bjorl.2021.07.001.
73. Lo, W.-C.; Wu, C.-T.; Lee, H.C.; Young, Y.-H.; Chang, Y.-L.; Cheng, P.-W. Evaluation of geranylgeranylacetone against cisplatin-induced ototoxicity by auditory brainstem response, heat shock proteins and oxidative levels in guinea pigs. *Neurotoxicol. Teratol.* **2017**, *61*, 29–35, doi:10.1016/j.ntt.2017.03.004.
74. Choi, S.J.; Kim, S.W.; Lee, J.B.; Lim, H.J.; Kim, Y.J.; Tian, C.; So, H.S.; Park, R.; Choung, Y.-H. Gingko biloba extracts protect auditory hair cells from cisplatin-induced ototoxicity by inhibiting perturbation of gap junctional intercellular communication. *Neuroscience* **2013**, *244*, 49–61, doi:10.1016/j.neuroscience.2013.04.001.
75. Esen, E.; Özdoğan, F.; Gürgen, S.G.; Özel, H.E.; Başer, S.; Genç, S.; Selçuk, A. Ginkgo biloba and Lycopene are Effective on Cisplatin Induced Ototoxicity? *J. Int. Adv. Otol.* **2018**, *14*, 22–26, doi:10.5152/iao.2017.3137.
76. Huang, X.; Whitworth, C.A.; Rybak, L.P. Ginkgo biloba extract (EGb 761) protects against cisplatin-induced ototoxicity in rats. *Otol. Neurotol.* **2007**, *28*, 828–833, doi:10.1097/mao.0b013e3180430163.
77. Xu, O.; Lu, H.; Li, P.; Zhang, X.; Lu, Z. Effect of combination of Ginkgo leaf extract and deferoxamine in preventing and treating ototoxicity of cisplatin. *Zhongguo Zhong Xi Yi Jie He Za Zhi* **2004**, *24*, 915–918.
78. Fei, B.; Liu, Z.; Xie, L.; Lv, L.; Zhu, W.; Liu, J.; Dai, Y.; She, W. Panax notoginseng Saponins protect auditory cells against cisplatin-induced ototoxicity by inducing the AKT/Nrf2 signaling-mediated redox pathway. *Mol. Med. Rep.* **2020**, *22*, 3533–3540, doi:10.3892/mmr.2020.11390.
79. Im, G.J.; Chang, J.W.; Choi, J.; Chae, S.W.; Ko, E.J.; Jung, H.H. Protective effect of Korean red ginseng extract on cisplatin ototoxicity in HEI-OC1 auditory cells. *Phytother. Res.* **2010**, *24*, 614–621, doi:10.1002/ptr.3082.
80. Qiao, X.; He, Y.; Li, W.; Liu, C.; Yang, J.; Li, H. 20(S)-Ginsenoside Rh1 inhibits cisplatin-induced hearing loss by inhibiting the MAPK signaling pathway and suppressing apoptosis in vitro. *Biochim. Biophys. Acta Mol. Cell Res.* **2023**, *1870*, 119461, doi:10.1016/j.bbamcr.2023.119461.
81. Lou, J.; Wu, F.; He, W.; Hu, R.; Cai, Z.; Chen, G.; Zhao, W.; Zhang, Z.; Si, Y. Hesperidin activates Nrf2 to protect cochlear hair cells from cisplatin-induced damage. *Redox Rep.* **2024**, *29*, 2341470, doi:10.1080/13510002.2024.2341470.
82. Kara, M.; Türkön, H.; Karaca, T.; Güçlü, O.; Uysal, S.; Türkyılmaz, M.; Demirtaş, S.; Dereköy, F.S. Evaluation of the protective effects of hesperetin against cisplatin-induced ototoxicity in a rat animal model. *Int. J. Pediatr. Otorhinolaryngol.* **2016**, *85*, 12–18, doi:10.1016/j.ijporl.2016.03.019.
83. Kikkawa, Y.S.; Nakagawa, T.; Taniguchi, M.; Ito, J. Hydrogen protects auditory hair cells from cisplatin-induced free radicals. *Neurosci. Lett.* **2014**, *579*, 125–129, doi:10.1016/j.neulet.2014.07.025.

84. Qu, J.; Li, X.; Wang, J.; Mi, W.; Xie, K.; Qiu, J. Inhalation of hydrogen gas attenuates cisplatin-induced ototoxicity via reducing oxidative stress. *Int. J. Pediatr. Otorhinolaryngol.* **2012**, *76*, 111–115, doi:10.1016/j.ijporl.2011.10.014.
85. Zhang, W.; Man, R.; Yu, X.; Yang, H.; Yang, Q.; Li, J. Hydroxytyrosol enhances cisplatin-induced ototoxicity: Possible relation to the alteration in the activity of JNK and AIF pathways. *Eur. J. Pharmacol.* **2020**, *887*, 173338, doi:10.1016/j.ejphar.2020.173338.
86. Bekmez Bilmez, Z.E.; Aydin, S.; Şanli, A.; Altintoprak, N.; Demir, M.G.; Atalay Erdoğan, B.; Kösemihal, E. Oxytocin as a protective agent in cisplatin-induced ototoxicity. *Cancer Chemother. Pharmacol.* **2016**, *77*, 875–879, doi:10.1007/s00280-016-2978-x.
87. Celebi, S.; Gurdal, M.M.; Ozkul, M.H.; Yasar, H.; Balikci, H.H. The effect of intratympanic vitamin C administration on cisplatin-induced ototoxicity. *Eur. Arch. Otorhinolaryngol.* **2013**, *270*, 1293–1297, doi:10.1007/s00405-012-2140-2.
88. Wang, W.; Qiu, S.; Zhang, T.; Zheng, Z.; Zhu, K.; Gao, X.; Zhao, F.; Ma, X.; Lin, H.; He, Y.; et al. Quantum chemistry calculation-aided discovery of potent small-molecule mimics of glutathione peroxidases for the treatment of cisplatin-induced hearing loss. *Eur. J. Med. Chem.* **2024**, *271*, 116404, doi:10.1016/j.ejmech.2024.116404.
89. Alan, M.A.; Eryılmaz, M.A.; Kaymaz, F.; Suzer, A.; Arıcıgil, M. Isotretinoin's action against cisplatin-induced ototoxicity in rats. *Pak. J. Pharm. Sci.* **2018**, *31*, 2579–2584.
90. Lee, H.-S.; Kim, Y.-R.; Lee, I.-K.; Kim, U.-K.; Baek, J.-I.; Lee, K.-Y. KL1333, a derivative of β-lapachone, protects against cisplatin-induced ototoxicity in mouse cochlear cultures. *Biomed. Pharmacother.* **2020**, *126*, 110068, doi:10.1016/j.biopha.2020.110068.
91. Nader, M.-E.; Théorêt, Y.; Saliba, I. The role of intratympanic lactate injection in the prevention of cisplatin-induced ototoxicity. *Laryngoscope* **2010**, *120*, 1208–1213, doi:10.1002/lary.20892.
92. Abd-Allah, A.R.A.; Al-Majed, A.A.; Al-Yahya, A.A.; Fouda, S.I.; Al-Shabana, O.A. L-Carnitine halts apoptosis and myelosuppression induced by carboplatin in rat bone marrow cell cultures (BMC). *Arch. Toxicol.* **2005**, *79*, 406–413, doi:10.1007/s00204-004-0643-3.
93. Gozeler, M.S.; Ekinci Akdemir, F.N.; Yildirim, S.; Sahin, A.; Eser, G.; Askin, S. Levosimendan ameliorates cisplatin-induced ototoxicity: Rat model. *Int. J. Pediatr. Otorhinolaryngol.* **2019**, *122*, 70–75, doi:10.1016/j.ijporl.2019.04.004.
94. Koo, D.Y.; Lee, S.H.; Lee, S.; Chang, J.; Jung, H.H.; Im, G.J. Comparison of the effects of lipoic acid and glutathione against cisplatin-induced ototoxicity in auditory cells. *Int. J. Pediatr. Otorhinolaryngol.* **2016**, *91*, 30–36, doi:10.1016/j.ijporl.2016.10.008.
95. Li, A.; He, Y.; Sun, S.; Cai, C.; Li, H. Lysine-specific demethylase 1 inhibitors protect cochlear spiral ganglion neurons against cisplatin-induced damage. *Neuroreport* **2015**, *26*, 539–547, doi:10.1097/WNR.0000000000000386.
96. Roldán-Fidalgo, A.; Martín Saldaña, S.; Trinidad, A.; Olmedilla-Alonso, B.; Rodríguez-Valiente, A.; García-Berrocal, J.R.; Ramírez-Camacho, R. In vitro and in vivo effects of lutein against cisplatin-induced ototoxicity. *Exp. Toxicol. Pathol.* **2016**, *68*, 197–204, doi:10.1016/j.etp.2016.01.003.
97. Ciçek, M.T.; Kalcioğlu, T.M.; Bayindir, T.; Toplu, Y.; Iraz, M. The effect of lycopene on the ototoxicity induced by cisplatin. *Turk. J. Med. Sci.* **2014**, *44*, 582–585, doi:10.3906/sag-1304-66.
98. Ozkırış, M.; Kapusuz, Z.; Karaçavuş, S.; Saydam, L. The effects of lycopene on cisplatin-induced ototoxicity. *Eur. Arch. Otorhinolaryngol.* **2013**, *270*, 3027–3033, doi:10.1007/s00405-013-2352-0.
99. Noman, A.; Mukherjee, S.; Le, T.N. Manipulating the blood labyrinth barrier with mannitol to prevent cisplatin-induced hearing loss. *Hear. Res.* **2022**, *426*, 108646, doi:10.1016/j.heares.2022.108646.
100. Kasse, C.A.; Cruz, O.L.M.; Iha, L.C.N.; Costa, H.O.; Lopes, E.C.; Coelho, F. The use of Maytenus ilicifolia to prevent cisplatin-induced ototoxicity. *Braz. J. Otorhinolaryngol.* **2008**, *74*, 712–717, doi:10.1016/S1808-8694(15)31381-1.

101. Araujo, J.G. de; Serra, L.S.M.; Lauand, L.; Kückelhaus, S.A.S.; Sampaio, A.L.L. Protective Effect of Melatonin on Cisplatin-induced Ototoxicity in Rats. *Anticancer Res.* **2019**, *39*, 2453–2458, doi:10.21873/anticanres.13364.
102. Assumpção, N.L.; Araújo, J.G. de; Serra, L.S.M.; Ribeiro, V.V.; Sampaio, M.L.Q.; Caram, A.A.; Sampaio, A.L.L. Effect of melatonin on otoprotection in rodents: a systematic review with meta-analysis. *Braz. J. Otorhinolaryngol.* **2023**, *89*, 101288, doi:10.1016/j.bjorl.2023.101288.
103. Demir, M.G.; Altıntoprak, N.; Aydın, S.; Kösemihal, E.; Başak, K. Effect of Transtympanic Injection of Melatonin on Cisplatin-Induced Ototoxicity. *J. Int. Adv. Otol.* **2015**, *11*, 202–206, doi:10.5152/iao.2015.1094.
104. Cappaert, N.L.M.; Klis, S.F.L.; Wijbenga, J.; Smoorenburg, G.F. Acceleration of cisplatin ototoxicity by perilymphatic application of 4-methylthiobenzoic acid. *Hear. Res.* **2005**, *203*, 80–87, doi:10.1016/j.heares.2004.10.012.
105. Doğan, M.; Polat, H.; Yaşar, M.; Kaya, A.; Bayram, A.; Şenel, F.; Özcan, İ. Protective role of misoprostol against cisplatin-induced ototoxicity. *Eur. Arch. Otorhinolaryngol.* **2016**, *273*, 3685–3692, doi:10.1007/s00405-016-4031-4.
106. Tate, A.D.; Antonelli, P.J.; Hannabass, K.R.; Dirain, C.O. Mitochondria-Targeted Antioxidant Mitoquinone Reduces Cisplatin-Induced Ototoxicity in Guinea Pigs. *Otolaryngol. Head Neck Surg.* **2017**, *156*, 543–548, doi:10.1177/0194599816678381.
107. Bentli, R.; Parlakpinar, H.; Polat, A.; Samdanci, E.; Sarihan, M.E.; Sagir, M. Molsidomine prevents cisplatin-induced hepatotoxicity. *Arch. Med. Res.* **2013**, *44*, 521–528, doi:10.1016/j.arcmed.2013.09.013.
108. Toplu, Y.; Parlakpinar, H.; Sapmaz, E.; Karatas, E.; Polat, A.; Kizilay, A. The protective role of molsidomine on the Cisplatin-induced ototoxicity. *Indian J. Otolaryngol. Head Neck Surg.* **2014**, *66*, 314–319, doi:10.1007/s12070-014-0718-2.
109. Chen, B.-C.; Lin, L.-J.; Lin, Y.-C.; Lee, C.-F.; Hsu, W.-C. Optimal N-acetylcysteine concentration for intratympanic injection to prevent cisplatin-induced ototoxicity in guinea pigs. *Acta Otolaryngol.* **2022**, *142*, 127–131, doi:10.1080/00016489.2022.2038796.
110. Dickey, D.T.; Wu, Y.J.; Muldoon, L.L.; Neuwelt, E.A. Protection against cisplatin-induced toxicities by N-acetylcysteine and sodium thiosulfate as assessed at the molecular, cellular, and in vivo levels. *J. Pharmacol. Exp. Ther.* **2005**, *314*, 1052–1058, doi:10.1124/jpet.105.087601.
111. Kedeshian, K.; Hong, M.; Hoffman, L.; Kita, A. N-acetylcysteine Microparticles Reduce Cisplatin-induced RSC96 Schwann Cell Toxicity. *bioRxiv* **2023**, doi:10.1101/2023.10.31.564430.
112. Muldoon, L.L.; Wu, Y.J.; Pagel, M.A.; Neuwelt, E.A. N-acetylcysteine chemoprotection without decreased cisplatin antitumor efficacy in pediatric tumor models. *J. Neurooncol.* **2015**, *121*, 433–440, doi:10.1007/s11060-014-1657-1.
113. Okur, E.; Kilinc, M.; Yildirim, I.; Kilic, M.A.; Tolun, F.I. Effect of N-acetylcysteine on carboplatin-induced ototoxicity and nitric oxide levels in a rat model. *Laryngoscope* **2007**, *117*, 2183–2186, doi:10.1097/MLG.0b013e31813e6041.
114. Somdaş, M.A.; Güntürk, İ.; Balcıoğlu, E.; Avcı, D.; Yazıcı, C.; Özdamar, S. Protective effect of N-acetylcysteine against cisplatin ototoxicity in rats: a study with hearing tests and scanning electron microscopy. *Braz. J. Otorhinolaryngol.* **2020**, *86*, 30–37, doi:10.1016/j.bjorl.2018.08.002.
115. Wang, W.; Chen, E.; Ding, X.; Lu, P.; Chen, J.; Ma, P.; Lu, L. N-acetylcysteine protect inner hair cells from cisplatin by alleviated celluar oxidative stress and apoptosis. *Toxicol. In Vitro* **2022**, *81*, 105354, doi:10.1016/j.tiv.2022.105354.
116. Wu, Y.J.; Muldoon, L.L.; Neuwelt, E.A. The chemoprotective agent N-acetylcysteine blocks cisplatin-induced apoptosis through caspase signaling pathway. *J. Pharmacol. Exp. Ther.* **2005**, *312*, 424–431, doi:10.1124/jpet.104.075119.

117. Moon, I.J.; Kim, K.R.; Chu, H.-S.; Kim, S.H.; Chung, W.-H.; Cho, Y.-S.; Hong, S.H. N-acetylcysteine and N-nitroarginine methyl ester attenuate Carboplatin-induced ototoxicity in dissociated spiral ganglion neuron cultures. *Clin. Exp. Otorhinolaryngol.* **2011**, *4*, 11–17, doi:10.3342/ceo.2011.4.1.11.
118. Choe, W.-T.; Chinosornvatana, N.; Chang, K.W. Prevention of cisplatin ototoxicity using transtympanic N-acetylcysteine and lactate. *Otol. Neurotol.* **2004**, *25*, 910–915, doi:10.1097/00129492-200411000-00009.
119. Sooriyaarachchi, M.; Narendran, A.; Gailer, J. N-acetyl-L-cysteine modulates the metabolism of cis-platin in human plasma in vitro. *Metallomics* **2013**, *5*, 197–207, doi:10.1039/c3mt00012e.
120. Akaras, N.; Gür, C.; Caglayan, C.; Kandemir, F.M. Protective effects of naringin against oxaliplatin-induced testicular damage in rats: Involvement of oxidative stress, inflammation, endoplasmic reticulum stress, apoptosis, and histopathology. *Iran. J. Basic Med. Sci.* **2024**, *27*, 466–474, doi:10.22038/IJBMS.2024.73824.16048.
121. Li, M.; Liu, J.; Liu, D.; Duan, X.; Zhang, Q.; Wang, D.; Zheng, Q.; Bai, X.; Lu, Z. Naringin attenuates cisplatin- and aminoglycoside-induced hair cell injury in the zebrafish lateral line via multiple pathways. *J. Cell. Mol. Med.* **2021**, *25*, 975–989, doi:10.1111/jcmm.16158.
122. Dalian, D.; Haiyan, J.; Yong, F.; Yongqi, L.; Salvi, R.; Someya, S.; Tanokura, M. Ototoxic Model of Oxaliplatin and Protection from Nicotinamide Adenine Dinucleotide. *J. Otol.* **2013**, *8*, 63–71, doi:10.1016/s1672-2930(13)50009-2.
123. Song, W.; Zhang, L.; Cui, X.; Wang, R.; Ma, J.; Xu, Y.; Jin, Y.; Wang, D.; Lu, Z. Nobiletin alleviates cisplatin-induced ototoxicity via activating autophagy and inhibiting NRF2/GPX4-mediated ferroptosis. *Sci. Rep.* **2024**, *14*, 7889, doi:10.1038/s41598-024-55614-4.
124. Gao, X.; Mao, H.; Zhao, L.; Li, X.; Liao, Y.; Li, W.; Li, H.; Chen, Y. Nuciferine Protects Cochlear Hair Cells from Ferroptosis through Inhibiting NCOA4-Mediated Ferritinophagy. *Antioxidants (Basel)* **2024**, *13*, doi:10.3390/antiox13060714.
125. Youn, C.K.; Jo, E.-R.; Sim, J.-H.; Cho, S.I. Peanut sprout extract attenuates cisplatin-induced ototoxicity by induction of the Akt/Nrf2-mediated redox pathway. *Int. J. Pediatr. Otorhinolaryngol.* **2017**, *92*, 61–66, doi:10.1016/j.ijporl.2016.11.004.
126. Li, D.; Zhao, H.; Xu, P.; Lin, Q.; Zhao, T.; Li, C.; Cui, Z.-K.; Tian, G. Polydatin activates the Nrf2/HO-1 signaling pathway to protect cisplatin-induced hearing loss in guinea pigs. *Front. Pharmacol.* **2022**, *13*, 887833, doi:10.3389/fphar.2022.887833.
127. Yazici, Z.M.; Meric, A.; Midi, A.; Arınc, Y.V.; Kahya, V.; Hafiz, G. Reduction of cisplatin ototoxicity in rats by oral administration of pomegranate extract. *Eur. Arch. Otorhinolaryngol.* **2012**, *269*, 45–52, doi:10.1007/s00405-011-1582-2.
128. Xu, B.; Li, J.; Chen, X.; Kou, M. Puerarin attenuates cisplatin-induced apoptosis of hair cells through the mitochondrial apoptotic pathway. *Biochim. Biophys. Acta Mol. Cell Res.* **2022**, *1869*, 119208, doi:10.1016/j.bbamcr.2021.119208.
129. Gündoğdu, R.; Erkan, M.; Aydın, M.; Sönmez, M.F.; Vural, A.; Kökoğlu, K.; Karabulut, D.; Şahin, M.İ. Assessment of the Effectiveness of Quercetin on Cisplatin-Induced Ototoxicity in Rats. *J. Int. Adv. Otol.* **2019**, *15*, 229–236, doi:10.5152/iao.2019.5902.
130. Huang, T.-L.; Jiang, W.-J.; Zhou, Z.; Shi, T.-F.; Yu, M.; Yu, M.; Si, J.-Q.; Wang, Y.-P.; Li, L. Quercetin attenuates cisplatin-induced mitochondrial apoptosis via PI3K/Akt mediated inhibition of oxidative stress in pericytes and improves the blood labyrinth barrier permeability. *Chem. Biol. Interact.* **2024**, *393*, 110939, doi:10.1016/j.cbi.2024.110939.
131. Lee, S.K.; Oh, K.H.; Chung, A.Y.; Park, H.C.; Lee, S.H.; Kwon, S.Y.; Choi, J. Protective role of quercetin against cisplatin-induced hair cell damage in zebrafish embryos. *Hum. Exp. Toxicol.* **2015**, *34*, 1043–1052, doi:10.1177/0960327114567766.

132. Najafi, M.; Tavakol, S.; Zarrabi, A.; Ashrafizadeh, M. Dual role of quercetin in enhancing the efficacy of cisplatin in chemotherapy and protection against its side effects: a review. *Arch. Physiol. Biochem.* **2022**, *128*, 1438–1452, doi:10.1080/13813455.2020.1773864.
133. Alsaikhan, F.; Jasim, S.A.; Margiana, R.; Opulencia, M.J.C.; Yasin, G.; Hammid, A.T.; Nasretdinova, M.T.; Mahdi, A.B.; Farhood, B.; Abedi-Firouzjah, R.; et al. Recent update on the protective potentials of resveratrol against cisplatin-induced ototoxicity: a systematic review. *Curr. Med. Chem.* **2023**, doi:10.2174/0929867331666230724124013.
134. Erdem, T.; Bayindir, T.; Filiz, A.; Iraz, M.; Selimoglu, E. The effect of resveratrol on the prevention of cisplatin ototoxicity. *Eur. Arch. Otorhinolaryngol.* **2012**, *269*, 2185–2188, doi:10.1007/s00405-011-1883-5.
135. Lee, C.H.; Kim, K.W.; Lee, S.M.; Kim, S.Y. Dose-Dependent Effects of Resveratrol on Cisplatin-Induced Hearing Loss. *Int. J. Mol. Sci.* **2020**, *22*, doi:10.3390/ijms22010113.
136. Lee, S.H.; Kim, H.S.; An, Y.S.; Chang, J.; Choi, J.; Im, G.J. Protective effect of resveratrol against cisplatin-induced ototoxicity in HEI-OC1 auditory cells. *Int. J. Pediatr. Otorhinolaryngol.* **2015**, *79*, 58–62, doi:10.1016/j.ijporl.2014.11.008.
137. Liu, Y.; Wu, H.; Zhang, F.; Yang, J.; He, J. Resveratrol upregulates miR-455-5p to antagonize cisplatin ototoxicity via modulating the PTEN-PI3K-AKT axis. *Biochem. Cell Biol.* **2021**, *99*, 385–395, doi:10.1139/bcb-2020-0459.
138. Olgun, Y.; Kırkım, G.; Kolatan, E.; Kıray, M.; Bagrıyanık, A.; Olgun, A.; Kızmazoglu, D.C.; Ellıdokuz, H.; Serbetcıoglu, B.; Altun, Z.; et al. Friend or foe? Effect of oral resveratrol on cisplatin ototoxicity. *Laryngoscope* **2014**, *124*, 760–766, doi:10.1002/lary.24323.
139. Simşek, G.; Tokgoz, S.A.; Vuralkan, E.; Caliskan, M.; Besalti, O.; Akin, I. Protective effects of resveratrol on cisplatin-dependent inner-ear damage in rats. *Eur. Arch. Otorhinolaryngol.* **2013**, *270*, 1789–1793, doi:10.1007/s00405-012-2183-4.
140. Yumusakhuylu, A.C.; Yazici, M.; Sari, M.; Binnetoglu, A.; Kosemihal, E.; Akdas, F.; Sirvanci, S.; Yuksel, M.; Uneri, C.; Tutkun, A. Protective role of resveratrol against cisplatin induced ototoxicity in guinea pigs. *Int. J. Pediatr. Otorhinolaryngol.* **2012**, *76*, 404–408, doi:10.1016/j.ijporl.2011.12.021.
141. Üstün Bezgin, S.; Uygur, K.K.; Gökdoğan, Ç.; Elmas, Ç.; Göktaş, G. The Effects of Riluzole on Cisplatin-induced Ototoxicity. *Int. Arch. Otorhinolaryngol.* **2019**, *23*, e267-e275, doi:10.1055/s-0038-1676654.
142. Munguia, R.; Sahmkow, S.I.; Funnell, W.R.J.; Daniel, S.J. Transtympanic Ringer's lactate application in the prevention of cisplatinum-induced ototoxicity in a chinchilla animal model. *Otolaryngol. Head Neck Surg.* **2010**, *143*, 134–140, doi:10.1016/j.otohns.2010.02.007.
143. Zheng, Z.; Wang, Y.; Yu, H.; Li, W.; Wu, J.; Cai, C.; He, Y. Salvianolic acid B inhibits ototoxic drug-induced ototoxicity by suppression of the mitochondrial apoptosis pathway. *J. Cell. Mol. Med.* **2020**, *24*, 6883–6897, doi:10.1111/jcmm.15345.
144. Li, Y.; Liu, Z.; Chen, J.; Wang, R.; An, X.; Tian, C.; Yang, H.; Zha, D. Schisandrin B protect inner hair cells from cisplatin by inhibiting celluar oxidative stress and apoptosis. *Toxicol. In Vitro* **2024**, *99*, 105852, doi:10.1016/j.tiv.2024.105852.
145. Doğan, S.; Yazici, H.; Yalçinkaya, E.; Erdoğdu, H.I.; Tokgöz, S.A.; Sarici, F.; Namuslu, M.; Sarikaya, Y. Protective Effect of Selenium Against Cisplatin-Induced Ototoxicity in an Experimental Design. *J. Craniofac. Surg.* **2016**, *27*, e610-e614, doi:10.1097/SCS.0000000000002942.
146. Cho, S.I.; Lee, J.-E.; Do, N.Y. Protective effect of silymarin against cisplatin-induced ototoxicity. *Int. J. Pediatr. Otorhinolaryngol.* **2014**, *78*, 474–478, doi:10.1016/j.ijporl.2013.12.024.
147. Hyppolito, M.A.; de Oliveira, José Antonio A; Rossato, M. Cisplatin ototoxicity and otoprotection with sodium salicylate. *Eur. Arch. Otorhinolaryngol.* **2006**, *263*, 798–803, doi:10.1007/s00405-006-0070-6.

148. Pierre, P.V.; Engmér, C.; Wallin, I.; Laurell, G.; Ehrsson, H. High concentrations of thiosulfate in scala tympani perilymph after systemic administration in the guinea pig. *Acta Otolaryngol.* **2009**, *129*, 132–137, doi:10.1080/00016480802116232.
149. Youn, C.K.; Kim, J.; Jo, E.-R.; Oh, J.; Do, N.Y.; Cho, S.I. Protective Effect of Tempol against Cisplatin-Induced Ototoxicity. *Int. J. Mol. Sci.* **2016**, *17*, doi:10.3390/ijms17111931.
150. Kuduban, O.; Kucur, C.; Sener, E.; Suleyman, H.; Akcay, F. The role of thiamine pyrophosphate in prevention of cisplatin ototoxicity in an animal model. *ScientificWorldJournal.* **2013**, *2013*, 182694, doi:10.1155/2013/182694.
151. Sagit, M.; Korkmaz, F.; Akcadag, A.; Somdas, M.A. Protective effect of thymoquinone against cisplatin-induced ototoxicity. *Eur. Arch. Otorhinolaryngol.* **2013**, *270*, 2231–2237, doi:10.1007/s00405-012-2254-6.
152. Di, Y.; Xu, T.; Tian, Y.; Ma, T.; Qu, D.; Wang, Y.; Lin, Y.; Bao, D.; Yu, L.; Liu, S.; et al. Ursolic acid protects against cisplatin-induced ototoxicity by inhibiting oxidative stress and TRPV1-mediated Ca2+-signaling. *Int. J. Mol. Med.* **2020**, *46*, 806–816, doi:10.3892/ijmm.2020.4633.
153. Kim, S.K.; Im, G.J.; An, Y.S.; Lee, S.H.; Jung, H.H.; Park, S.Y. The effects of the antioxidant α-tocopherol succinate on cisplatin-induced ototoxicity in HEI-OC1 auditory cells. *Int. J. Pediatr. Otorhinolaryngol.* **2016**, *86*, 9–14, doi:10.1016/j.ijporl.2016.04.008.
154. Özdemir, D.; Özgür, A.; Kalkan, Y.; Terzi, S.; Tümkaya, L.; Yılmaz, A.; Çeliker, M.; Dursun, E. The protective effects of whortleberry extract against cisplatin-induced ototoxicity in rats. *Braz. J. Otorhinolaryngol.* **2019**, *85*, 55–62, doi:10.1016/j.bjorl.2017.10.009.
155. Lee, C.H.; Lee, D.-H.; Lee, S.M.; Kim, A.S.Y. Otoprotective Effects of Zingerone on Cisplatin-Induced Ototoxicity. *Int. J. Mol. Sci.* **2020**, *21*, doi:10.3390/ijms21103503.
